# Innovative Nanotechnology in Drug Delivery Systems for Advanced Treatment of Posterior Segment Ocular Diseases

**DOI:** 10.1002/advs.202403399

**Published:** 2024-06-21

**Authors:** Yue Wu, Xin Li, Xueyu Fu, Xiaomin Huang, Shenrong Zhang, Nan Zhao, Xiaowei Ma, Qimanguli Saiding, Mei Yang, Wei Tao, Xingtao Zhou, Jinhai Huang

**Affiliations:** ^1^ Eye Institute and Department of Ophthalmology, Eye & ENT Hospital, Fudan University; NHC Key Laboratory of Myopia and Related Eye Diseases; Key Laboratory of Myopia and Related Eye Diseases Chinese Academy of Medical Sciences Shanghai 200031 China; ^2^ Shanghai Research Center of Ophthalmology and Optometry Shanghai 200031 China; ^3^ Wenzhou Medical University Wenzhou Zhejiang 325035 China; ^4^ Center for Nanomedicine and Department of Anesthesiology Brigham and Women's Hospital, Harvard Medical School Boston MA 02115 USA

**Keywords:** anti‐VEGF therapy, drug delivery, nanomedicine, posterior segment ocular diseases, ROS scavenging

## Abstract

Funduscopic diseases, including diabetic retinopathy (DR) and age‐related macular degeneration (AMD), significantly impact global visual health, leading to impaired vision and irreversible blindness. Delivering drugs to the posterior segment of the eye remains a challenge due to the presence of multiple physiological and anatomical barriers. Conventional drug delivery methods often prove ineffective and may cause side effects. Nanomaterials, characterized by their small size, large surface area, tunable properties, and biocompatibility, enhance the permeability, stability, and targeting of drugs. Ocular nanomaterials encompass a wide range, including lipid nanomaterials, polymer nanomaterials, metal nanomaterials, carbon nanomaterials, quantum dot nanomaterials, and so on. These innovative materials, often combined with hydrogels and exosomes, are engineered to address multiple mechanisms, including macrophage polarization, reactive oxygen species (ROS) scavenging, and anti‐vascular endothelial growth factor (VEGF). Compared to conventional modalities, nanomedicines achieve regulated and sustained delivery, reduced administration frequency, prolonged drug action, and minimized side effects. This study delves into the obstacles encountered in drug delivery to the posterior segment and highlights the progress facilitated by nanomedicine. Prospectively, these findings pave the way for next‐generation ocular drug delivery systems and deeper clinical research, aiming to refine treatments, alleviate the burden on patients, and ultimately improve visual health globally.

## Graphical abstract

The revolutionary nanotechnology brings hope to posterior segment ocular diseases. This review delves into the challenges of conventional drug delivery and the advantages of diverse nanomaterials in enhancing drug stability, targeting, and permeability by targeting VEGF, ROS, macrophages. Focusing on the latest developments, this paper examines nanomedicine's potential to transform therapeutic approaches and improve global visual health.

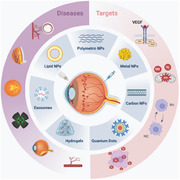

## 1 Introduction

Funduscopic diseases include a variety of conditions in the vitreous, choroid, and retina, causing structural and functional abnormalities in the eye. Among these, there is a significant increase in the prevalence of diabetic retinopathy (DR) and age‐related macular degeneration (AMD). This is mainly attributed to the growing incidence of diabetes mellitus, the aging population, and other lifestyle factors. These diseases significantly impact visual experience and overall quality of life of patients, posing a severe public health challenge.^[^ ^1^ ^]^

The medications routinely used to manage posterior segment disorders primarily include anti‐vascular endothelial growth factor (Anti‐VEGF) treatments, such as conbercept, along with glucocorticoids and antioxidants.^[^ ^2^ ^]^ The most common treatment for treating ocular conditions is through topical application of eye drops. However, these drugs often encounter difficulty in penetrating the deeper tissue layers at the lesion site because of the physiological and anatomical obstacles that exist within the eye.^[^ ^3^ ^]^ Alternative approaches like periocular and intraocular injections can more directly target the affected area and offer prolonged drug action.^[^ ^4^ ^]^ However, these methods come with their drawbacks, including pain, as well as risks of infection and bleeding.^[^ ^5^ ^]^ The blood‐retina barrier hinders effective drug delivery to the target site despite the practicality and common usage of systemic drug administration. Furthermore, long‐term application of some substances, such as corticosteroids, may result in rising intraocular pressure as well as multiple side effects.

**Figure 1:**
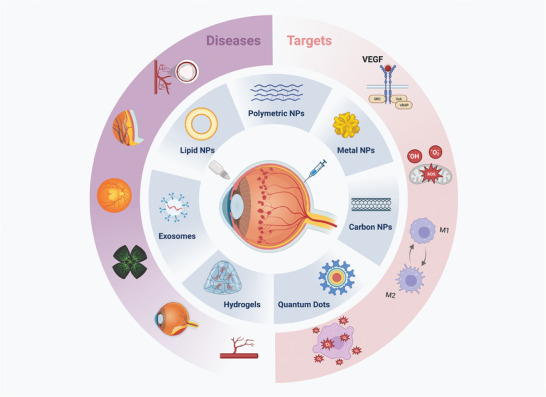
Multiple fabricated nanomaterials against different targets for the treatment of posterior segment ocular diseases. Created with BioRender.com.

Nanotechnology has led to the creation of innovative drug delivery methods using nanoparticles for treating posterior segment diseases. Nanoparticles (NPs), first and foremost, offer the potential for targeted drug delivery.^[^ ^6^ ^]^ They can be engineered with precision to selectively target tissues or cells, including photoreceptor cells or the retinal pigment epithelium (RPE). Moreover, nanocarriers help to minimize side effects by safeguarding the drug against degradation in vivo, enhancing absorption and distribution, and increasing drug bioavailability, thus reducing the required dosage for effective treatment. Nanomedicines can prolong drug release, increasing the therapeutic impact lifetime and perhaps eliminating the requirement for frequent dosage, which can significantly improve patient adherence. In addition, these nanomedicines can carry various therapeutic ingredients such as small‐molecule drugs, biologics, and genes. They provide non‐invasive delivery options such as eye drops or ointments, while also serving imaging functionalities.^[^ ^7^ ^]^ Nevertheless, the biosafety, stability, and manufacturing processes of these nanomaterials require further exploration and refinement. **Figure** **1** illustrates a diagram outlining the treatment of posterior ocular disorders using nanomaterials. This review explores the intricacies and challenges of administering medications to the posterior segment of the eye, focusing on the prominent advancements and improvements that nanomedicine has made in treating disorders affecting the fundus. These achievements signify a crucial progression, establishing the foundation for innovative breakthroughs in the field of ocular medication delivery systems.

## 2 Principal Posterior Segment Ocular Diseases and the Pathophysiological Mechanisms

The eyeball is a nearly spherical structure, with the anterior 1/6 being the transparent cornea and the remaining 5/6 being the sclera. Behind the sclera lies the optic nerve, which connects to the intracranial visual center. The eye is conventionally conceived as a composite of two integrated units: the smaller anterior segment, which encompasses the cornea, iris, lens, anterior chamber, and ciliary body, whereas the larger posterior segment consists of the retina, choroid, vitreous humor, and among others. Disorders of the posterior segment represent a spectrum of conditions characterized by structural and functional aberrations in the vitreous, retina, and choroid. Predominant posterior segment diseases, such as DR, AMD, retinal vein occlusion, pathologic myopia, retinitis pigmentosa, uveitis, and retinoblastoma, are increasingly impacting populations globally, culminating in profound visual impairments and irreversible blindness.^[^ ^8^ ^]^ These disorders share several common pathophysiological mechanisms, including involvement of VEGF, reactive oxygen species (ROS) and oxidative stress, macrophages, RPE damage, mitochondrial dysfunction, and matrix metalloproteinases. A comprehensive understanding of these fundamental variables is crucial for clarifying the development of diseases affecting the posterior part of the eye, paving the way for the implementation of advanced and reliable treatments that prioritize safety and efficacy.

### 2.1 Major Posterior Ocular Segment Diseases and Treatments

In the rapidly advancing realm of medicine today, gaining a comprehensive understanding of ophthalmic disorders has become progressively vital. Notably, posterior ocular diseases, particularly AMD and DR, are emerging as significant threats to both the visual and systemic health of patients.

#### 2.1.1 Age‐Related Macular Degeneration

AMD is a degenerative eye disease that mostly affects vision in older individuals. As individuals get older, the likelihood of experiencing visual impairment increases. This makes AMD the primary reason for irreversible blindness in individuals aged 50 and above.^[^ ^9^ ^]^ In light of an aging global population and lifestyle changes, the prevalence of AMD is anticipated to surge, with projections suggesting that 1 in 10 Americans over 50 years old will be diagnosed with AMD by 2050.^[^ ^10^ ^]^ AMD is categorized into two types: dry AMD and wet AMD (wAMD). Dry AMD, also known as atrophic AMD, is the more prevalent form among patients. It typically progresses gradually over several years as the macula thins along with aging. On the other hand, wet AMD, also known as advanced neovascular AMD, is a rarer form of advanced AMD but often leads to more rapid vision loss or even total loss. Choroidal neovascularization stands out as a significant characteristic of wAMD. Notably, VEGF has been established as a key player in the pathogenesis of wet AMD. The main manifestations of AMD include visual distortions, diminished central visual acuity or the presence of dark spots, reduced contrast sensitivity, and visual field deficits. AMD is a multifactorial disease, with its pathogenesis involving an array of interrelated factors such as lipid peroxidation, RPE retinal pigment epithelial cell senescence, inflammation and oxidative stress, neovascularization, and mitochondrial damage.^[^ ^11^ ^]^ In the initial stages, RPE metabolism is disturbed by ROS and lipids. This disruption leads to the accumulation of metabolite fragments and proteins, thickening the Bruch's membrane. Subsequently, there is an imbalance in the levels of pro‐angiogenic and anti‐angiogenic substances in the tissue. Neovascularization, originating in the choroid, extends subretinally, severely impacting vision and potentially leading to irreversible visual impairment.

Promising approaches for AMD include anti‐neovascular therapies (including anti‐VEGF medications, laser treatments, and surgical options), inhibition of the complement and other inflammatory pathways, reduction of oxidative stress, suppression of lipofuscin and optic cycling, as well as innovative methods for atrophic AMD like RPE cell transplantation and stem cell regenerative therapy.^[^ ^12^ ^]^ Prophylactic therapy utilizing antioxidants such as vitamin C, vitamin E, and beta carotene, in addition to statins, is indicated for patients in the early stages of AMD. Interventions aimed at dry AMD primarily focus on postponing its progression to wet AMD, involving the utilization of antioxidants like vitamin C, vitamin E, and beta carotene, alongside statins. Therapeutic strategy development for wet AMD predominantly focuses on anti‐choroidal neovascularization(anti‐CNV) therapy. Commonly employed treatments in such cases include anti‐VEGF drugs, laser photocoagulation, photodynamic therapy, radiotherapy, transpupillary thermotherapy, and various modalities of macular surgery.^[^ ^13^ ^]^

#### 2.1.2 Diabetic Retinopathy

DR, a persistent and incapacitating consequence of diabetes, has become the primary reason for visual impairment in the global population of adults who are of working age.^[^ ^14^ ^]^ DR affects about thirty percent of people with diabetes, and approximately 15% have different stages of retinopathy when they are first diagnosed with diabetes.^[^ ^15^ ^]^ Notably, the rate of blindness in diabetic patients is a staggering 25 times higher than that of non‐diabetic individuals, underscoring the urgent need for effective prevention and treatment strategies.^[^ ^16^ ^]^ Beyond the devastating impact on patients' visual health, DR also imposes a considerable economic burden on the public health system.

The progression of DR is intrinsically linked to prolonged hyperglycemic states, with the severity of its lesions closely associated with the duration of diabetes. Hyperglycemia triggers a series of metabolic pathway irregularities, including upregulation of the polyol pathway and formation of advanced glycation end‐products. These alterations induce oxidative stress and provoke inflammatory reactions, resulting in heightened vascular permeability and dysfunction of the blood‐retinal barrier. This results in leakage from the central retinal artery, ciliary vessels, and deeper retinal layers, thereby triggering ischemic occlusions in capillaries and compensatory neovascular formation and proliferation. Clinically, based on the findings from the multicenter Early Treatment Diabetic Retinopathy Study (ETDRS), DR is divided into two types: non‐proliferative diabetic retinopathy (NPDR) and proliferative diabetic retinopathy (PDR). This categorization is based on whether there are observable changes in the eye, particularly retinal neovascularization.^[^ ^17^ ^]^

The cornerstone of managing DR is rigorous glycemic control, which can significantly reduce the risk of retinopathy. When fundus microcirculation is compromised, argon laser photocoagulation is recommended to improve retinal circulation. Intravitreal injections of anti‐VEGF medicines are effective in treating macular edema and improving vision in cases with pre‐proliferative or proliferative diabetic retinopathy.^[^ ^18^ ^]^ In advanced DR patients experiencing intravitreal hemorrhage and neovascularization, vitrectomy can be performed as a preventative measure against vision loss and visual field defects.^[^ ^19^ ^]^ While these treatment modalities have been widely adopted in clinical practice, their effectiveness remains suboptimal in some patients, and they are not without limitations. These include patient discomfort, low adherence, long‐term side effects, high economic costs, and other potential risks. Hence, it is imperative to promptly develop new therapeutic targets and techniques to overcome these obstacles.

#### 2.1.3 Retinal Vein Occlusion

Retinal vein occlusion (RVO) is a prevalent retinal vascular disorder resulting from the blockage of venous reflux in the retina, typically affecting older individuals. RVO can be classified into three forms depending on the site of the obstruction: central retinal vein occlusion (CRVO), hemi‐retinal vein occlusion (HRVO), and branch retinal vein occlusion (BRVO).^[^ ^20^ ^]^ Common symptoms of RVO include blurred vision, decreasing visual acuity, and distorted or asymmetrical vision. The vision loss experienced by individuals with RVO can be caused by retinal hemorrhage, ischemia and neovascularization, vitreous hemorrhage, and principally macular edema. However, it is crucial to realize that macular edema is the main factor leading to vision impairment in these patients.^[^ ^21^ ^]^ Contemporary therapeutic approaches for RVO include steroidal ocular implants, laser photocoagulation, and anti‐VEGF injections. Although these modalities are effective, they are constrained by potential adverse effects, including local irritation, hemorrhage, and infection. Hence, it is vital to discover new treatment targets and ways to address the challenges associated with RVO.

#### 2.1.4 Pathological Myopia

High myopia (HM), characterized by a refractive error exceeding −6.0 D, can progress to involve significant changes in the retina and choroid. This advanced stage, known as pathological myopia (PM), encompasses a spectrum of ocular alterations, including leopard‐spot fundus, choroidal retinal atrophy, posterior scleral staphyloma, lacquer cracks, macular splitting, macular hemorrhage, subretinal neovascularization, and retinal detachment.^[^ ^22^ ^]^

Choroidal neovascularization secondary to pathological myopia (mCNV) is a major contributor to severe vision impairment in young and middle‐aged adults worldwide, particularly among Asian populations.^[^ ^23^ ^]^ In contrast to CNV in AMD, mCNV predominantly occurs between the retinal photoreceptors and the RPE layer, whereas AMD‐related neovascularization typically develops in the sub‐RPE interstitial space.^[^ ^24^ ^]^ Historically, laser photocoagulation (LP) was a primary treatment modality for mCNV, but its application has diminished due to limited efficacy, high recurrence rates, and substantial complication risks. Intravitreal injections of anti‐VEGF drugs have emerged as the leading approach for managing macular choroidal neovascularization mCNV, establishing a new benchmark in care.^[^ ^25^ ^]^

#### 2.1.5 Glaucoma

Glaucoma refers to a range of eye disorders that are characterized by the degeneration of the optic nerve and the emergence of visual field defects. It is currently the second most prevalent cause of blindness globally and the primary cause of permanent blindness.^[^ ^26^ ^]^ The most crucial risk factor is an abnormally high intraocular pressure (IOP) that is pathological. Obstruction in the flow of fluid in the anterior part of the eye can cause an elevation in IOP, which can then lead to injury of the optic nerve located in the rear part of the eye.

Surgery is an effective method for treating glaucoma, with common procedures including trabeculectomy, iridectomy, and ciliary body cryo/photo‐coagulation. The primary pharmacotherapy for glaucoma comprises medications that reduce IOP through various mechanisms. These include beta‐blockers such as timolol, parasympathomimetics like pilocarpine, prostaglandin analogs, and carbonic anhydrase inhibitors. Although IOP‐lowering therapies can effectively slow disease progression in some patients, they may not prevent optic nerve damage or vision loss, particularly in patients with normal IOP. Ongoing research on neuroprotective therapies for glaucoma demonstrates promise, aiming to treat the disease by preventing the death or degeneration of retinal ganglion cells (RGC).^[^ ^27^ ^]^ Nonetheless, to date, no neuroprotective treatments have been officially approved for clinical application. This category includes many kinds of antioxidants,^[^ ^28^ ^]^ such as N‐methyl‐d‐aspartate (NMDA) receptor antagonists, calcium channel blockers, aspirin, melatonin, and Vitamin B12.^[^ ^29^ ^]^

#### 2.1.6 Retinitis Pigmentosa

Retinitis Pigmentosa (RP) is a genetic retinal degenerative illness that is chronic and progressive. It is the primary cause of inherited blindness globally, affecting around 1 in 4000 individuals. Clinically, RP initially presents with night blindness and a progressive, centripetal constriction of the visual field. In its advanced stages, patients may experience “tunnel vision” and ultimately complete blindness. Pathologically, RP is distinguished by the degeneration of retinal photoreceptor cells (rods and cones) by both apoptotic and non‐apoptotic pathways. The primary factors contributing to this degeneration include oxidative stress, dysregulation of energy metabolism, abnormalities in autophagy, and immunological responses.^[^ ^30^ ^]^ Current therapeutic strategies are primarily aimed at symptomatic relief and delaying disease progression. These include the administration of vitamin A, docosahexaenoic acid (DHA), lutein, steroids, and anti‐VEGF agents, along with emerging interventions such as gene therapy, stem cell transplantation, and artificial retina implantation, which hold promise for future RP management.^[^ ^31^ ^]^

#### 2.1.7 Uveitis

The uvea, an intermediate layer in the eye's structure, comprises of a vascular‐rich loose connective tissue abundant in pigment. Uveitis, a condition posing a threat to vision, is characterized by inflammation affecting the eye. This inflammation can result in consequences like retinal detachment, glaucoma, cataracts, and other severe vision abnormalities. There are two types of uveitis: infectious and non‐infectious, with the latter mainly consisting of autoimmune uveitis. Currently, the clinical management of uveitis poses a significant challenge. Standard treatment regimens primarily include glucocorticoids, immunosuppressive agents, and biologic therapies, notably tumor necrosis factor (TNF) inhibitors like adalimumab.^[^ ^32^ ^]^ However, these pharmacological interventions often carry substantial toxic side effects and may not consistently yield satisfactory therapeutic outcomes in all patients. Therefore, the pursuit of safer and more efficacious treatment strategies remains a crucial focus in uveitis research.

#### 2.1.8 Retinoblastoma

Retinoblastoma (RB), originating in the retina, is the most prevalent primary intraocular malignant eye tumor in children and is relatively rare in adults. It has a global incidence of ≈ 1 in 15 000 to 1 in 20 000 and is classified into heritable and non‐heritable forms. Approximately two‐thirds of cases initially present unilaterally. Clinical manifestations include a cataract‐like white pupillary reflex, strabismus, vision loss, nystagmus, and ocular redness. The tumor may extend extraocularly through the sclera or intracranially along the optic nerve. The current therapeutic approach for RB includes chemotherapy, radiotherapy, surgical intervention, laser therapy, and cryotherapy. Furthermore, emerging treatments such as gene therapy,^[^ ^33^ ^]^ targeted therapy, and immunotherapy are offering new hope.^[^ ^34^ ^]^

### 2.2 Pathophysiological Mechanisms

The distinct pathophysiological mechanisms of posterior ocular diseases not only represent a key area of medical research but also present a critical challenge that requires urgent attention in clinical practice. This section delves into the underlying mechanisms and theories of disease occurrence are elucidated, serving as the foundation and potential aims for the development of effective therapeutic substances.

#### 2.2.1 Vascular Endothelial Growth Factor

Angiogenesis is the biological mechanism via which new blood vessels develop from pre‐existing ones. It is crucial for embryonic development, tumor growth, and the progression of certain eye illnesses. It involves a series of complex molecular regulatory mechanisms.^[^ ^35^ ^]^ In 1989, VEGF was first successfully isolated and cloned from cultured bovine pituitary follicular cells, significantly advancing the understanding of the pathophysiological process of angiogenesis.^[^ ^36^ ^]^ VEGF is a family of proteins that regulate angiogenesis, increase vascular permeability, modify extracellular matrix, and stimulate endothelial cell proliferation, migration, and lumen formation. This family includes VEGFA, VEGFB, VEGFC, VEGFD, VEGFE, and Placental Growth Factor (PlGF, also known as PGF). Given its dominant role in regulating angiogenesis and disease, VEGF‐A is often abbreviated as VEGF. VEGFA transcripts form precursor mRNA that, through alternative splicing, can produce various isoforms, including VEGF121, VEGF145, VEGF165, and VEGF189. Under the influence of driving factors such as Hypoxia‐Inducible Factors (HIF), VEGF is expressed and binds to tyrosine kinase receptors VEGFR1, VEGFR2, and VEGFR3 on the endothelial cell membrane. This interaction leads to the autophosphorylation of these receptors, activating the Mitogen‐Activated Protein Kinase (MAPK) pathway and stimulating endothelial cell growth.^[^ ^37^ ^]^ Additionally, VEGF can also increase the fragility of existing functional blood vessels and lead to leakage.

#### 2.2.2 Reactive Oxygen and Nitrogen Species (RONS) and Oxidative Stress

ROS are produced chemicals by aerobic cells during metabolism, essentially the monoelectronic reduction products of oxygen compounds. In mitochondria, electron transfer chain complexes pass electrons to O_2_, leading to the generation of these highly reactive by‐products due to incomplete oxygen reduction.^[^ ^38^ ^]^ Major components of ROS include the superoxide anion (O_2_•^−^), hydrogen peroxide (H_2_O_2_), singlet oxygen (^1^O_2_), and the hydroxyl radical (•OH). Reactive nitrogen species (RNS) mainly consist of nitrites (NO_2_^−^), nitrates (NO_3_^−^), and peroxynitrites (ONOO^−^).^[^ ^39^ ^]^ RONS influence on the regulation of diverse physiological processes in the human body substantially. Under normal conditions, the production and clearance of ROS in the body are in a dynamic equilibrium. Enzymes such as superoxide dismutase (SOD), glutathione peroxidase (GSH), and glutathione S‐transferase (GST) within tissues can effectively neutralize the damage induced by ROS. However, due to anomalies such as lipid metabolism and photodamage, the tissue's ability to remove excessive reactive oxygen and nitrogen free radicals is overwhelmed, resulting in oxidative stress. This leads to DNA, lipid, and protein damage, causing irreversible toxic effects on cells and triggering cell apoptosis. The outer segment membranes of retinal photoreceptors, which contain a high amount of polyunsaturated fatty acids, are especially vulnerable to oxidative harm. In diseases such as AMD, intense oxidative stress leads to the accumulation of lipids in RPE cells, disrupting normal cellular function and metabolism. This accumulation of metabolic products on the Bruch's membrane creates a barrier to oxygen diffusion, further inducing neovascularization.

#### 2.2.3 Macrophages

Macrophages (Mf) are a crucial type of immune cell capable of engulfing and eliminating foreign bodies, pathogens, and dead cells. In the eye, macrophages are involved in various physiological processes and exist in almost all ocular tissues. Emerging studies suggest that macrophages have a crucial regulatory function in the ocular illness progression. They contribute to tissue damage and repair, regulate inflammatory responses,^[^ ^40^ ^]^ stimulate the formation of new blood vessels, and are a key element in the pathological process of neovascularization.^[^ ^41^ ^]^ Macrophages can differentiate into different polarities under the stimulation of various tissue microenvironments, primarily categorized as classically activated (M1 type) and alternatively activated (M2 type).^[^ ^42^ ^]^ M1 type, activated by lipopolysaccharides (LPS) or interferon‐gamma (IFN‐γ), can increase vascular permeability. On the other hand, M2 type, activated by anti‐inflammatory factors, TH2 cell cytokines, or glycoproteins, can inhibit angiogenesis. Macrophages can release a multitude of cytokines that promote the formation of neovascularization. The M1 type produces inflammatory cytokines such as TNF‐α, IL‐12, IL‐23, IL‐1β, and IL‐6, playing a role in inflammatory responses. In contrast, the M2 type secretes anti‐inflammatory agents, including IL‐10, TGF‐β, IL‐1R II, and IL‐1Ra.^[^ ^43^ ^]^

#### 2.2.4 RPE Damage and Mitochondrial Dysfunction

RPE, located between the photoreceptors in the retina and the choroid, consists of a single layer of hexagonal cells rich in pigment granules and forms a crucial metabolic interface, performing functions such as light absorption, substance transport, immune response, and secretion. It is essential for maintaining the homeostasis and normal function of the retina.^[^ ^44^ ^]^ Damage and dysfunction of the RPE are considered crucial elements in the development of posterior segment disorders like AMD. The RPE, acting as the metabolic center of the retina, is distinguished by its high density and activity of mitochondria. It is responsible for phagocytizing, digesting, and renewing photoreceptor outer segments, as well as for material exchange between the retina and the choroid. However, with aging, mitochondrial structural and functional abnormalities gradually emerge in RPE cells, such as mitochondrial DNA (mtDNA) damage and mutations, reduced oxidative phosphorylation, increased ROS, and impaired mitophagy. In a controlled study, Terluk et al. observed that mtDNA damage in the RPE of AMD patients significantly increased, while there was no change in mtDNA damage in the neural retina.^[^ ^45^ ^]^ These changes affect the metabolic, clearance, and antioxidative capacities of RPE cells, promoting the development of AMD.

#### 2.2.5 Matrix Metalloproteinases

Matrix metalloproteinases (MMPs) are a group of calcium‐dependent, zinc‐containing enzymes that can degrade several protein elements of the extracellular matrix, including gelatin, collagen, elastin, and fibronectin. These enzymes facilitate the secretion of bioactive substances, such as growth factors and apoptotic ligands, on the cell surface and participate in the cleavage of cell surface receptors.^[^ ^46^ ^]^ Their activities have a significant impact on crucial cellular processes including proliferation, migration, differentiation, apoptosis, and host defense.^[^ ^47^ ^]^ Consequently, MMPs play a vital role in the pathology of various diseases.^[^ ^48^ ^]^ The gelatinases MMP‐2 and MMP‐9, which are the most well‐researched and widely recognized members of the MMP family, possess the capacity to break down type IV collagen, a crucial constituent of the basement membrane. These enzymes have been associated with fostering the infiltration of cancer cells and their metastasis in various cancers, and recent studies have discovered a significant correlation between them and ocular neovascular diseases.^[^ ^49^ ^]^ Using a laser‐induced CNV model in mice, Lambert et al. compared CNV formation in mice with MMP‐2, MMP‐9, or dual gene deficiencies to that in control mice.^[^ ^50^ ^]^ The results indicated that MMP‐2 and MMP‐9 have a synergistic effect in CNV. The incidence and severity of CNV were significantly reduced in mice with both gene deficiencies, while mice with a single gene deficiency exhibited only a partial inhibitory effect. This phenomenon may be attributed to the accumulation of fibrin caused by the absence of MMP‐2 and MMP‐9, which hindered the progression of neovascularization.

## 3 Nanomaterials for Drug Delivery to the Posterior Ocular Segment

The eye's sophisticated structure, complex metabolic physiology, and unique natural barriers pose considerable obstacles to delivering drugs to the eye, setting it apart from other organs. The structure of the normal eye wall comprises three layers, progressing from external to internal. The outermost layer consists of collagen fiber tissue, formed by the transparent cornea at the front merging seamlessly with the milky white sclera at the back, creating a complete and closed outer wall that serves both a supportive and protective role for the eye's contents. The intermediate layer, the uvea, is rich in blood vessels and pigment, encompassing the iris, ciliary body, and choroid. At the innermost lies the retina, a complex and delicate structure that converts optical signals into neural signals. Delivering medications to the anterior part of the eye faces inherent obstacles, such as the tear film, cornea, conjunctiva, sclera, and lens.^[^ ^51^ ^]^ These obstacles are compounded when targeting the posterior portion, especially the retina, necessitating the navigation of additional specific anatomical structures and metabolic barriers. Conventional drug delivery methods approach frequently fail to attain optimal concentrations in target tissues, leading to suboptimal therapeutic outcomes. However, the rapid advancements in nanotechnology have significantly broadened the horizons for ocular drug delivery. A diverse array of nanocarrier drugs, encompassing lipid nanomaterials, polymer nanomaterials, metal and metal compound nanomaterials, carbon nanomaterials, quantum dot nanomaterials, and so on, are being explored. These innovative delivery devices are specifically engineered to circumvent these obstacles and have opened up new possibilities for treating eye illnesses in the posterior region. The primary objective of current research is to improve the administration of drugs to the eye by addressing these obstacles, ensuring the targeted distribution of therapeutic amounts of drug molecules to specified tissues in the posterior part of the eye with minimum side effects.^[^ ^52^ ^]^

### 3.1 Drug Delivery Routes to the Posterior Segment of the Eye

The three primary methods for delivering medications to the back part of the eye are topical application, systemic administration, and intravitreal injection. Other approaches also encompass subconjunctival, intracameral, and scleral administration.

#### 3.1.1 Topical Administration

Topical administration is often the preferred method for treating ocular diseases due to its simplicity. Eye drops serve as the main mode of local administration, with other forms including emulsions, ointments, suspensions, and contact lenses. The primary routes through which topically administered drugs enter the eye involve absorption through the cornea and conjunctiva, followed by transfer into the anterior chamber. While drugs in the anterior segment reach high concentrations, they often achieve lower concentrations in the posterior segment, making it challenging to reach effective therapeutic levels. Two primary strategies can be employed to improve the bioavailability of drugs in the eye post‐local administration: increasing the pre‐corneal retention time and enhancing the permeability of drugs through the cornea, sclera, and conjunctiva with low permeation.^[^ ^53^ ^]^ The advancement of nanoscale carriers can improve the ability of medicines to traverse the ocular surface, prolong the duration of drug interaction with ocular tissues, and deliver drugs to the posterior segment of the eye in a controlled manner, thus augmenting the quantity of available medication.

#### 3.1.2 Systemic Administration

Systemic therapies involve intravenous injection and oral administration. However, due to the constraints imposed by the blood‐retinal barrier, systemic delivery necessitates notably elevated medication dosages to breach the ocular obstacles. Once the drug reaches a therapeutically effective minimum concentration in the eye, its systemic concentration may already be high enough to cause toxicity and damage to other tissues and organs in the body. Therefore, the safety and biocompatibility of systemic drug administration are often disappointing. Artificially altering the blood‐retinal barrier can enhance drug permeability, but this approach is seldom used due to the potential for unnecessary side effects resulting from increased vascular permeability.

#### 3.1.3 Intravitreal Injection

Intravitreal injection is a commonly used method for treating vitreoretinal diseases. This approach allows for the direct administration of drugs into the vitreous body, avoiding the anterior segment components of the eye. It attains elevated therapeutic concentrations in the vitreous and retina while minimizing systemic absorption. However, being an invasive mode of drug delivery, it can lead to a less comfortable treatment experience and consequently cause lower patient adherence. Complications such as infection, inflammation, cataract formation, retinal tears, retinal detachment, and vitreous hemorrhage, either short or long‐term, limit its clinical application.^[^ ^54^ ^]^ Nanocarriers can enable precise and prolonged medication release, improving the effectiveness of intravitreal injection and extending its presence in the vitreous body. This significantly decreases the frequency of required injections and mitigates adverse effects. Moreover, there are ongoing efforts to produce different types of vitreous implants.^[^ ^55^ ^]^

### 3.2 Posterior Ocular Segment Drug Delivery Barriers

#### 3.2.1 Anatomical Barriers

**Figure 2:**
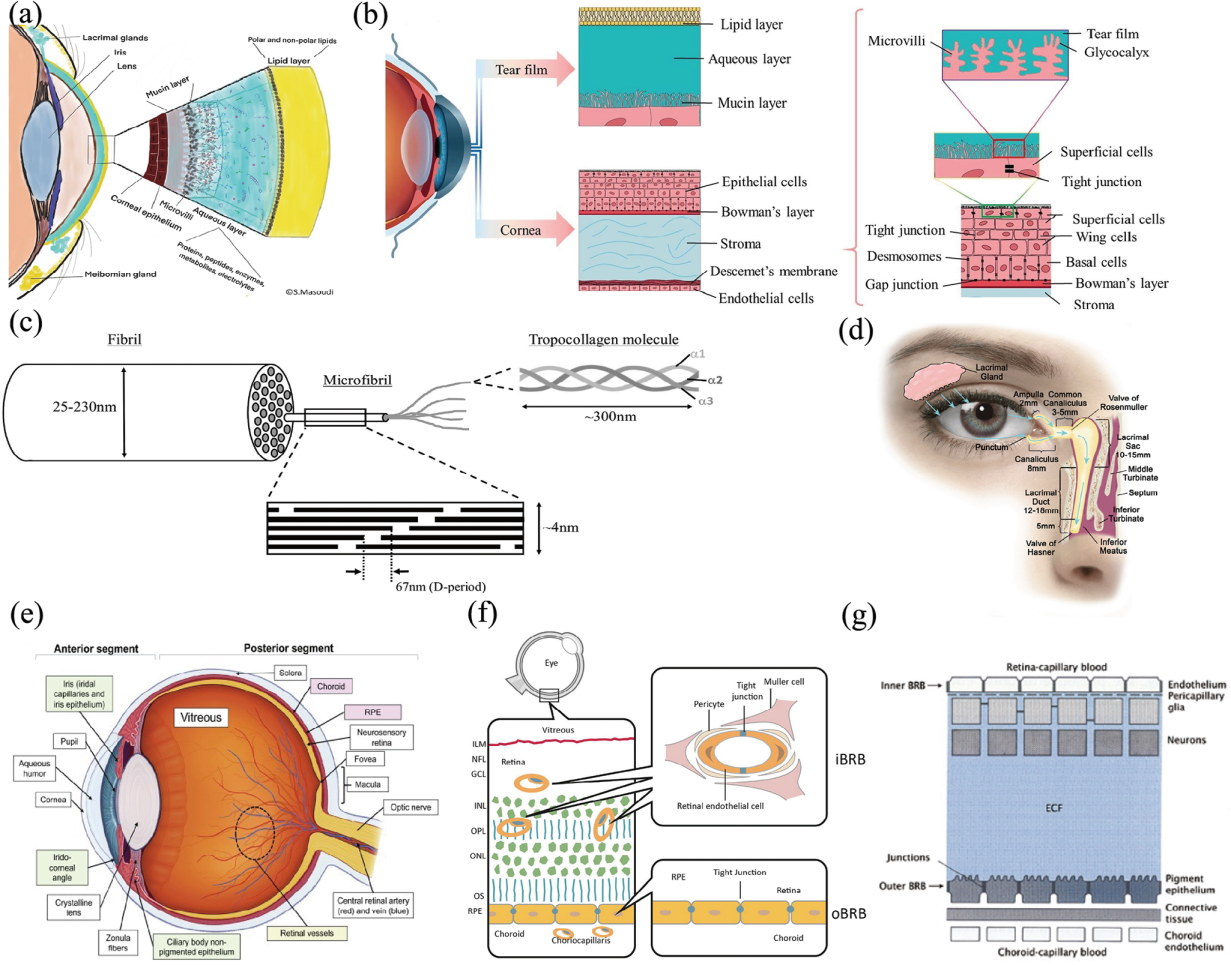
Ocular drug delivery barriers. a) Graphic representation of the three‐layered structure of the tear film, including the lipid layer, aqueous layer, and mucus layer. Reproduced with permission.^[^ ^56^ ^]^ Copyright 2022, Elsevier. b) The structure of the cornea reveals the presence of the corneal epithelium, stroma, and Descemet's membrane, and diagram depicting the tight junction of the corneal epithelium. Reproduced with permission.^[^ ^59^ ^]^ Copyright 2021, Elsevier. c) Schematic of the hierarchical collagen structure of the sclera. Reproduced with permission. Five triple alpha‐chain tropocollagen molecules assemble into microfibrils, in which the axial stagger of individual molecules leads to gap/overlap regions that define the 67 nm axial D‐period. Varying numbers of near‐parallel microfibrils form collagen fibrils of diameters ranging from 25 to 230 nm.^[^ ^63^ ^]^ Copyright 2020, Elsevier. d) Illustration of the lacrimal drainage system. Reproduced with permission.^[^ ^65^ ^]^ Copyright 2020, Elsevier. e) Anatomy of the human eye. The structures responsible for maintaining the blood‐aqueous barrier, the outer blood‐retinal barrier formed by the RPE, and the inner blood‐retinal barrier are color‐coded as green, pink, and yellow, respectively. Reproduced with permission.^[^ ^68^ ^]^ Copyright 2020, Elsevier. f) Anatomical localization of the inner and outer blood‐retinal barriers. Reproduced with permission.^[^ ^69^ ^]^ Copyright 2021, Wiley. g) Schematic presentation of the inner and outer blood‐retinal barriers and their relative location. ECF = extracellular fluid. Reproduced with permission.^[^ ^70^ ^]^ Copyright 2011, Wichtig Publishing Srl.

The tear film, cornea, and conjunctiva serve as primary barriers to the topical administration. The tear film is a liquid layer that covers the front surface of the eyeball and consists of three distinct layers: an outer lipid layer, a middle aqueous layer, and an inner mucin layer (**Figure** **2a**).^[^ ^56^ ^]^ It can affect the effective penetration of drugs through various mechanisms such as lipophilicity/hydrophilicity, protein binding and degradation, electrostatic repulsion,^[^ ^57^ ^]^ and drainage through the nasolacrimal duct.^[^ ^58^ ^]^ The cornea can be histologically divided into five layers, arranged from front to back: the layer of epithelial cells, Bowman's membrane, the stromal matrix, Descemet's membrane, and the layer of endothelial cells, and the corneal epithelium is hydrophobic and has strong intercellular connections that restrict the passage of large molecules (Figure 2b).^[^ ^59^ ^]^ Consequently, only small lipophilic molecules can cross the epithelium through transcellular transport. The corneal stroma, ≈500 µm thick, comprises ≈200 layers of collagen fiber lamellae and allows the diffusion of hydrophilic molecules within 500 kDa.^[^ ^60^ ^]^ The conjunctiva is a transparent mucous membrane that envelops the inner surface of the eyelids and extends to the anterior part of the eyeball. It consists of three parts: the palpebral conjunctiva, the bulbar conjunctiva, and the transitional fornix. Rich in nerves, blood vessels, and lymphatic tissue, the conjunctiva facilitates drug clearance through the circulatory and lymphatic systems, thus reducing ocular bioavailability.

The primary barriers in the posterior part of the eye include the sclera and vitreous. Due to its larger surface area,^[^ ^61^ ^]^ high hydration level, and lack of enzymes and protein binding sites,^[^ ^62^ ^]^ the sclera typically demonstrates greater permeability to drugs than the cornea, favoring drug permeation (Figure 2c).^[^ ^63^ ^]^ The vitreous, a transparent, gelatinous substance consisting mainly of water (98%) along with small amounts of collagen, hyaluronic acid, and proteins (0.15%), occupies the vitreous chamber and encompasses approximately four‐fifths of the eye's volume. It adheres to both the retina and the ciliary body. It acts as a significant diffusion barrier, especially for high molecular weight compounds in injection therapies.^[^ ^64^ ^]^ Hydrophilic and large molecular weight drugs exhibit an extended half‐life within the vitreous.

#### 3.2.2 Physiological Barriers

Drugs delivered using eye drops must first traverse the tear film and are prone to elimination through multiple processes. The most common processes include drug outflow due to blinking and elimination via the nasolacrimal duct pathway (Figure 2d).^[^ ^65^ ^]^ The turnover of tear fluid refers to the duration it remains on the eye surface. Commonly, the turnover rate of tear fluid ranges from 0.5 to 2.2 µL min^−1^. Post administration blinking reflexes accelerate this turnover rate, shortening the drug retention time on the eye surface. Tear fluid is secreted via a pathway starting from the lacrimal punctum to the lacrimal canaliculi, then to the lacrimal sac, and finally reaches the lower nasal meatus through the nasolacrimal duct. Studies indicate that the volume of a single eye drop is ≈50–60 µL, and if instilled slowly without blinking, the maximum volume that can be accommodated is up to 30 µL. The majority of the drug instilled into the conjunctiva, ≈80%−90%, is expelled through the nasolacrimal duct, with less than 5% of the drug entering the interior of the eye.^[^ ^66^ ^]^ Additionally, the properties of the drug and its formulation (pKa, molecular weight, preservatives, etc.) also influence its dynamic elimination. Mucins, primarily distributed over the conjunctiva and cornea, provide protection to the eye but simultaneously limit the absorption of drugs and affect the diffusion of large molecular drugs. Efflux transport proteins are also involved in the process of medication penetration and absorption.

#### 3.2.3 Blood‐Retinal Barrier

The blood‐retinal barrier (BRB), which consists of an inner and an outer barrier,^[^ ^67^ ^]^ serves as a crucial hindrance to the absorption of molecules into the eye when delivered through the scleral or systemic routes (Figure 2e).^[^ ^68^ ^]^ The inner BRB is comprised of the retinal capillary endothelium and its junctions, whereas the outer blood‐retinal barrier is constituted by the RPE (Figure 2f).^[^ ^69^ ^]^ The RPE separates the retinal tissue fluid from the choroidal fluid and, apart from its selective permeability, possesses the capability for active transport (Figure 2g).^[^ ^70^ ^]^ The BRB is essential for maintaining stability within the eye and ensuring the proper visual function of the retina. However, it also hinders the entry of medication molecules from the circulation system to the intended intraocular target areas.

### 3.3 Emerging Nanomaterials and Drug Delivery Platform for Ocular Diseases

#### 3.3.1 Lipid Nanomaterials

**Figure 3:**
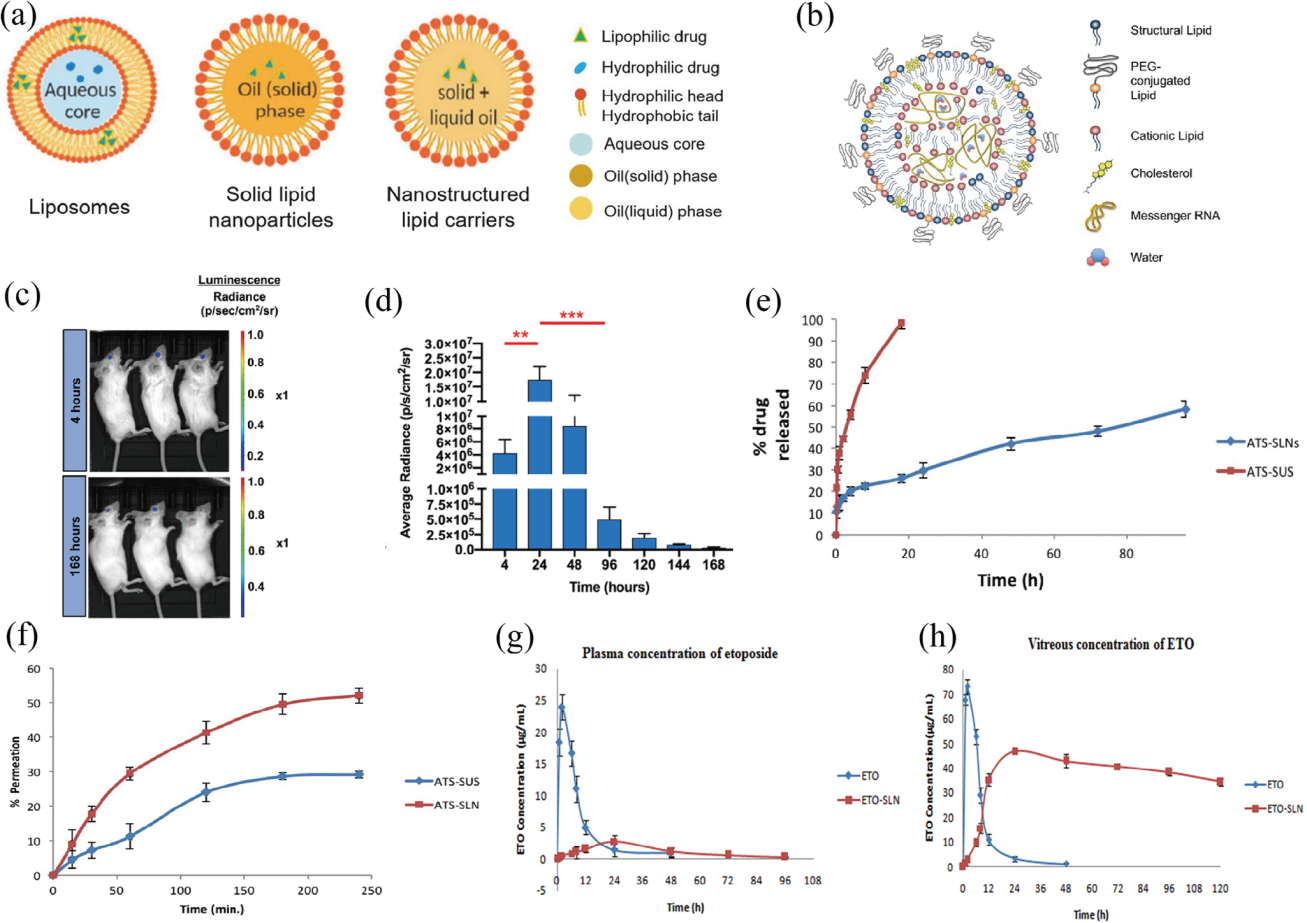
Lipid nanomaterials for ocular drug delivery. a) Diagram illustrates the structural composition of various types of lipid‐based nanoparticles, including liposomes, solid lipid nanoparticles (SLNs), and nanostructured lipid carriers. Reproduced under terms of the CC‐BY license.^[^ ^71^ ^]^ Copyright 2020, published by MDPI. b) Schematic structure of LNPs encapsulating mRNA and c) Images depicting bioluminescence at 24 and 168 h and d) Bar graph displays the quantified expression, measured as average radiance, at different time points after subretinal injections of MC3 LNPs with a dosage of 400 ng mRNA per injection. *n*  =  5; mean ± SEM. ** *p* ≤ 0.01, *** *p* ≤ 0.001. Reproduced with permission.^[^ ^82^ ^]^ Copyright 2019, Elsevier. e) The drug release of ATS‐SLNs and ATS‐SUS was evaluated in simulated tear fluid (pH 7.2) with the addition of 2% ethanol and f) The percentage of ATS‐SLNs and ATS‐SUS that passed through the porcine cornea at different time intervals (*n*  =  6). Reproduced with permission.^[^ ^86^ ^]^ Copyright 2020, Springer. g) Etoposide concentrations in plasma and h) vitreous after intravitreal injection of etoposide solution (ETO) and etoposide loaded SLNs (ETO‐SLNs) in rats (mean ± SD, *n*  =  3).^[^ ^90^ ^]^ Copyright 2019, Elsevier.

Lipid nanoparticles (LNPs), with particle diameters that range from 50 to 1000 nm and composed of lipids like mono/di‐/triglycerides, fatty acids, steroids, or waxes, exhibit enhanced biocompatibility and biosafety when compared to other nanomaterial types (**Figure** **3a**).^[^ ^71^ ^]^ Liposomes, characterized as spherical vesicles comprising one or more concentric layers of phospholipid bilayers, are distinguished by their non‐toxicity and biodegradability and have established themselves as efficacious drug delivery vehicles, extensively used within the realm of biomedical research. In 1990, M.R. Gasco and R.H. Müller pioneered an alternative to traditional nano‐systems, synthesizing what came to be known as solid lipid nanoparticles (SLNs) to distinguish them from nano‐emulsions and fluidic liposomes.^[^ ^72^ ^]^ In 1999, a new type of lipid nanoparticle nanostructured lipid carriers (NLCs) was developed.^[^ ^73^ ^]^ These NLCs are characterized by a matrix composed of both solid and liquid lipids. As integral members of this lipid nanoparticle family, liposomes, SLNs, and NLCs possess an oil‐in‐water (O/W) biphasic composition that improves the solubility of water‐insoluble drugs commonly employed in ophthalmic therapy. Their expansive specific surface area optimizes drug interaction and retention time within the eye. Moreover, by increasing the proportion of liquid lipid and employing diverse surfactants to achieve smaller particle sizes, drug penetration into the eye is improved. As a result, these lipid nanoparticles can be efficiently used in various ophthalmic applications with the aforementioned design.

##### Liposomes

Liposomes, typically ranging in diameter from 80 to 1000 nm, are structurally composed of single, double, or multiple concentric closed vesicles made up of lipid bilayers, which encapsulate an inner core that is hydrophilic in nature.^[^ ^74^ ^]^ Formed primarily from phospholipids, cholesterol, and wax lipids—components similar to those found in cell membranes—liposomes exhibit excellent biosafety and tissue compatibility. Their unique bilayer structure allows them to encapsulate both hydrophilic and hydrophobic medicines, efficiently delivering them to the specific site of action.^[^ ^75^ ^]^ In this arrangement, medications that are soluble in lipids can be incorporated into the interior of the lipid bilayer, while pharmaceuticals that are soluble in water can be dissolved in the inner watery core. The drug delivery efficacy of liposomes is intricately linked to their surface chemistry. Notably, the positive charge of liposomal nanodrugs facilitates binding to the negatively charged ocular surface, thereby enhancing drug action duration and increasing corneal permeability.^[^ ^76^ ^]^ These advantageous properties position liposomes as a highly promising carrier system for transporting drugs to the back of the eye.

In 1995, liposome‐based therapeutics, pioneering the realm of nanomedicine, received their inaugural approval from the US Food and Drug Administration. Since then, a wide range of commercially available liposomal products have been extensively used to treat a myriad of disorders affecting the posterior ocular segment, with a particular emphasis on AMD. An example is Visudyne, a photoactivatable liposomal formulation of verteporfin specifically indicated for the treatment of sub‐foveal choroidal neovascularization in AMD. Additionally, the intravitreal injection of liposomes loaded with tacrolimus has demonstrated efficacy in mitigating inflammatory responses, thereby offering a therapeutic approach for autoimmune uveitis.^[^ ^77^ ^]^

Altamirano‐Vallejo and colleagues developed and characterized a liposomal formulation specifically designed for ocular application, loaded with tretinoin (TA).^[^ ^78^ ^]^ The liposomal formulation provides a safer option compared to conventional intravitreal injections of steroidal preparations. In vivo analyses revealed that concentrations of TA in the retina and vitreous reached their maximum level 12 hours after the topical application. This delivery method effectively transported the drug to both the vitreous and retina without causing any significant impact on cell viability or intraocular pressure. Similar liposomal formulations have also been employed in the treatment of macular edema ME, a condition stemming from retinal capillary leakage. Li et al. devised a method to improve the effectiveness of delivering the long‐acting glucocorticoid tretinoin to the back part of the eye.^[^ ^79^ ^]^ They created a liposome that contains tretinoin and is encapsulated with chitosan, called TA‐CHL. The TA‐CHL formulation showed an average particle size of 135.46 ± 4.49 nm, along with excellent entrapment efficiency and the ability to release the drug gradually over time. Furthermore, it exhibited strong stability and minimal toxicity toward the cornea, conjunctiva, and retina. Gonzalez‐De la Rosa et al. conducted the latest exploratory clinical trial to evaluate the effectiveness of a liposomal tretinoin formulation with a concentration of 2 mg mL^−1^ in 12 patients who were experiencing refractory pseudocystic retinal edema.^[^ ^80^ ^]^ The treatment consisted of applying a single drop of this liposomal solution topically every 2 h for a period of 90 days. The study demonstrated a reduction in central foveal thickness (CFT) to 206.75 ± 135.72 µm and an enhancement in best‐corrected visual acuity (BCVA) to 20.08 ± 10.35 letters after 20 weeks of treatment.

Moreover, LNPs possess immense potential in RNA delivery, recently garnering widespread attention for their significant success in delivering COVID‐19 mRNA vaccines. Unshielded mRNA is inherently unstable and susceptible to destruction by nucleases. Encasing mRNA in LNPs shields it from external ribonucleases and aids in its intracellular transportation, making it a potent element for gene therapy in retinal disorders.^[^ ^81^ ^]^ Patel et al. measured the capability of 11 LNP variants mRNA delivery to the retina (Figure 3b).^[^ ^82^ ^]^ The most substantial levels of reporter gene transfection on the retina were observed in LNPs containing ionizable lipids characterized by low pKa values and unsaturated hydrocarbon chains. The onset of gene expression occurred promptly within 4 h, persisted for a prolonged period of up to 96 h, and exhibited selectivity toward RPE cells, rendering it appropriate for retinal reprogramming or genome editing (Figure 3c,d).^[^ ^82^ ^]^ This approach offers transformative new methods for treating monogenic retinal degenerative diseases of the RPE, potentially preventing blindness. Herrera‐Barrera et al. devised an LNP transporter directed by peptides to convey mRNA to the neural retina in rodents as well as primate species.^[^ ^83^ ^]^ Using a seven‐mer peptide phage display library based on the M13 bacteriophage, they identified peptides targeting photoreceptors (PRs). The LNPs, decorated with very effective peptide ligands, effectively transported mRNA to photoreceptor cells, RPE, and Müller glial cells in mice. This peptide‐guided LNP carrier demonstrated remarkable stability and efficient mRNA delivery capabilities, offering novel perspectives and tools for gene therapy in neuroretinal diseases.

##### Solid Lipid Nanoparticles

Researchers have shown considerable interest in solid lipid nanoparticles (SLNs) since the 1990s due to their potential use in medicine delivery. SLNs are colloidal nanoparticles ranging in size from 50 to 1000 nm. They are composed of a nonpolar solid core with a melting temperature higher than 40 °C, encased by surfactants in an aqueous solution.^[^ ^84^ ^]^ Their innovation lies in the replacement of liquid oils with solid lipids, including fatty acids, alcohols, glycerides, and waxes, to achieve controlled drug release. This is because drugs within solid lipids have significantly lower mobility and migration rates compared to those in liquid oils, offering substantial potential for enhancing drug performance and controlled release.^[^ ^85^ ^]^

SLNs offer numerous advantages, including enhanced drug load capacity, stability, and bioavailability, and protect fragile drugs from degradation. Yadav et al. developed an ATS‐SLNs eye drop formulation using a scalable thermal high‐pressure homogenization method (2339/DEL/2014) (Figure 3e,f).^[^ ^86^ ^]^ The availability of ATS‐SLNs in aqueous humor and vitreous humor was significantly greater when compared to free ATS, with increases of 8 and 12 times, respectively. Additionally, the corneal flux was 2.5 times higher, and stability was increased 13.62 times. Fluorescein labeling verified the successful administration of atorvastatin to the posterior segment. Furthermore, SLNs have also been extended to deliver microRNAs and functional genes for gene therapy in posterior segment eye diseases.^[^ ^87^ ^]^ The researchers led by M. Amadio employed SLNs to develop a nano‐system containing siRNA for the purpose of suppressing HuR expression (lipoplex).^[^ ^88^ ^]^ This nano‐system was then administered via injection into the eyes of diabetic rats induced by streptozotocin (STZ). Outcomes indicated a decrease in HuR and VEGF concentrations in the retinas of the treated cohort in comparison to the naked siRNA group, thereby highlighting its potential efficacy as a therapeutic intervention for retinal disorders.

SLNs facilitate controlled and targeted drug release while also exhibiting an attractive non‐biotoxicity profile. Isoniazid, a first‐line anti‐tuberculosis drug, often fails to achieve effective local concentrations when systemically administered for intraocular inflammations like choroiditis caused by Mycobacterium tuberculosis, resulting in multiple systemic side effects like hepatotoxicity and neurotoxicity. Singh et al. created an eye drop formulation consisting of INH‐SLN, which involved using SLNs tagged with fluorescein (F‐SLNs) for absorption trials.^[^ ^89^ ^]^ The INH‐SLNs demonstrated an extended duration of release of up to 48 hours, improved capacity to pass through the cornea (1.6 times), a five‐fold decrease in minimum inhibitory concentration (MIC), a 4.2‐fold increase in ocular bioavailability (AUC), and higher safety in terms of biological effects. Ahmad and colleagues used SLNs loaded with etoposide for posterior segment delivery in rats (Figure 3g,h).^[^ ^90^ ^]^ A single intravitreal administration sustained etoposide concentrations in the vitreous body for up to seven days without causing severe toxicity to the surrounding ocular tissues. These novel manufacturing strategies for etoposide‐loaded SLNs promise to improve the adverse reactions associated with traditional frequent intravitreal injections.

##### Nanostructured Lipid Carriers

Nanostructured lipid carriers (NLCs) are lipid nanoparticles formed by combining solid and liquid lipids at ambient temperature. This unique composition provides improved stability compared to previous generations of lipid nanoparticles.^[^ ^91^ ^]^ This unique mixture incorporates liquid lipids, effectively preventing the recrystallization of solid lipids during storage and improving the release and stability of the encapsulated drugs.^[^ ^92^ ^]^ Experimental results have confirmed that NLCs possess superior drug‐loading capabilities compared to SLNs. By combining liquid and solid lipids, the drug encapsulation efficiency can be significantly enhanced when creating SLNs.^[^ ^93^ ^]^

Du and colleagues successfully prepared NLCs, further incorporating triamcinolone acetonide (TA) for the posterior portion of the eye in mice through intraocular instillation.^[^ ^94^ ^]^ Stability analysis over a six‐month confirmed that the backscatter was less than 1.5%, indicating a reduced likelihood of nanoparticle aggregation and flocculation during storage.

#### 3.3.2 Polymeric Nanomaterials

Polymeric nanomaterials predominantly include compounds such as polylactic acid (PLA), poly (lactic‐co‐glycolic acid) (PLGA), collagen, chitosan, and gelatin. Due to their exceptional biocompatibility and biodegradability, these materials have been intensively researched and developed as basic matrix for the incorporation or encapsulation of medicinal medicines.^[^ ^95^ ^]^

PLA and PLGA have been used to create of diverse drug delivery methods, such as implants, contact lenses, and micelles. Zhang et al. used PLGA nanoparticles to encapsulate the anti‐VEGF monoclonal antibody Bevacizumab.^[^ ^96^ ^]^ The objective of this method was to prolong the retention of Bevacizumab in the vitreous body and aqueous humor. Consequently, it substantially improved the effectiveness of Bevacizumab in suppressing both corneal and retinal neovascularization. Additionally, PLGA‐encapsulated Bevacizumab enhances the bioavailability of the drug in ocular angiogenesis treatment and reduces its toxicity. Liu et al. used a solid/oil/water (S/O/W) emulsification technique to fabricate microspheres containing Bevacizumab, using PLGA/PCADK as the carrier material.^[^ ^97^ ^]^ The microspheres indicated a prolonged release pattern over a duration of 50 days, as observed in both laboratory settings and living organisms. They were well‐tolerated by ocular tissues, indicating their potential as a treatment technique for eye illnesses within the eye. Similarly, these materials have also been used for delivering drugs like dexamethasone, amikacin, and clindamycin.^[^ ^98^ ^]^ They have broad use in treating ocular illnesses like macular edema related to RVO, glaucoma,^[^ ^99^ ^]^ and others.^[^ ^100^ ^]^

**Figure 4:**
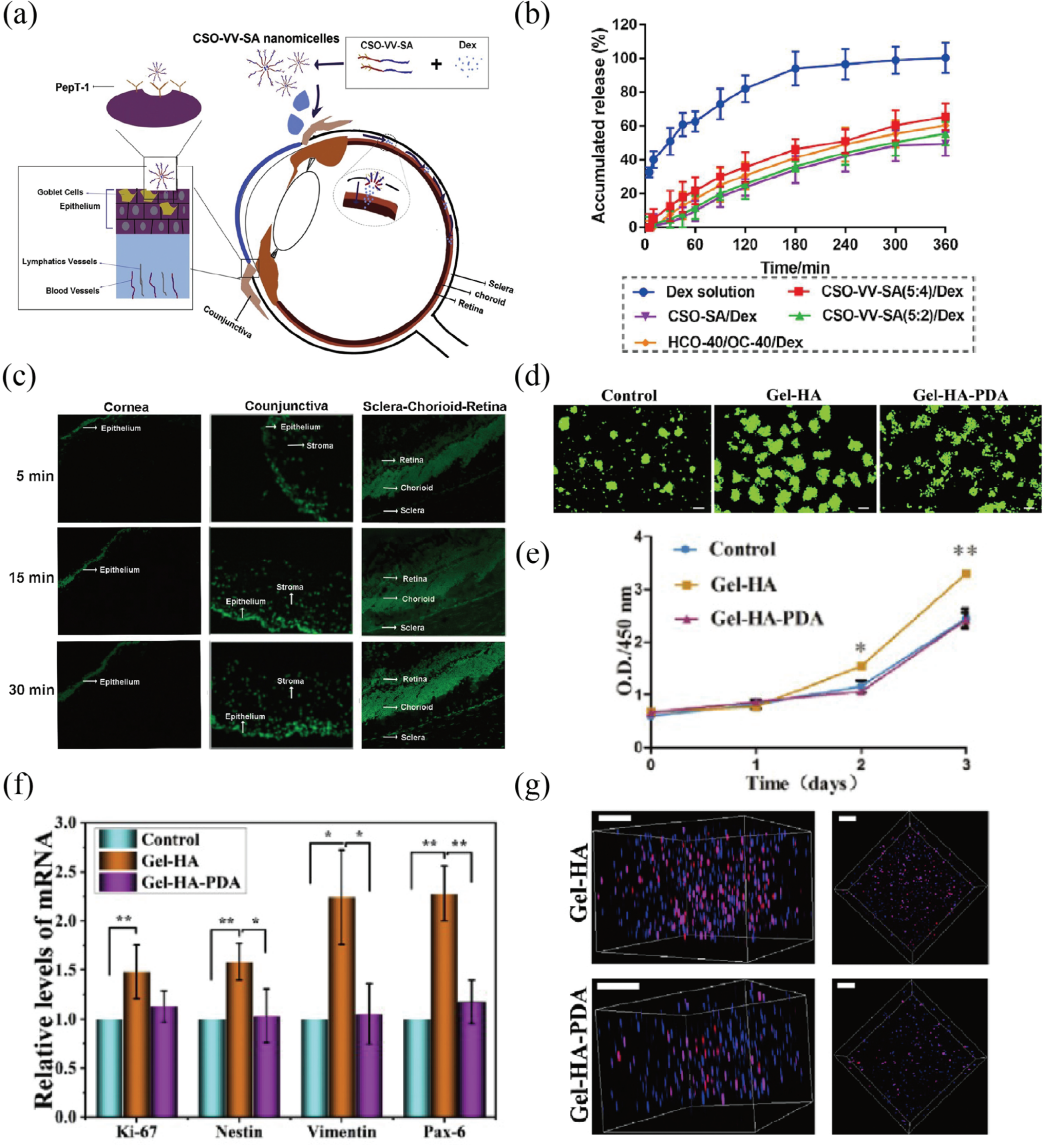
Polymeric nanomaterials. a–c) Chitosan oligosaccharide nanomicelles with functional properties are used to deliver dexamethasone to the ocular surface for topical medication administration. Reproduced with permission. a) Chitosan oligosaccharide‐valylvaline‐stearic acid nanomicelles were designed for topical ocular drug delivery, based on peptide transporter‐1 (PepT‐1) active targeting. b) In vitro drug release profiles of Dex solution, CSO‐SA nanomicelles, CSOVV‐SA (5:2) nanomicelles, CSO‐VV‐SA (5:4) nanomicelles, and HCO‐40/OC‐40/Dex mixed nanomicelles profiles in simulated tear fluid. c) Fluorescence microscopy of the cornea and conjunctiva of rabbit tissues, and sclera‐choroid‐retina of rat tissues after CSO‐VV‐SA (5:4)/Cou‐6 eyedrops administration. Reproduced with permission.^[^ ^102^ ^]^ Copyright 2020, Elsevier. d–g) Evaluation of RPC growth rate in relation to Gel‐HA and Gel‐HA‐PDA hydrogels following a three‐day incubation period. d) Fluorescent images of GFP‐positive retinal progenitor cells (RPCs) illustrated that the Gel‐HA hydrogel group had the largest concentration of cells. e) The growth potency of RPCs was significantly greater in the Gel‐HA, as evidenced by the CCK‐8 assay. f) The mRNA expression levels of the cell proliferation marker Ki‐67 and the retinal progenitor‐related markers Nestin, Vimentin, and Pax‐6 were significantly higher in cells cultured on Gel‐HA hydrogel compared to the control group. g) Edu staining of RPCs in 3D hydrogels. Reproduced with permission.^[^ ^106^ ^]^ Copyright 2019, Elsevier.

Chitosan (CH) is a naturally occurring hydrophilic cationic polysaccharide.^[^ ^101^ ^]^ Xu et al. developed a new branching nanoparticle composed of Chitosan Oligosaccharide‐Valine‐Valine‐Stearic Acid (CSO‐VV‐SA), which was designed to specifically target the peptide transporter‐1 (PepT‐1) for the delivery of dexamethasone (**Figure** **4a–c**).^[^ ^102^ ^]^ This formulation was specifically intended to access the posterior segment by utilizing the conjunctival route. In vivo tests conducted on male rats and male albino New Zealand rabbits demonstrated sustained release and enhanced permeation properties, comparable to the FDA‐approved nano‐emulsion blend of hydrogenated castor oil‐40/octanol‐40. Badiee et al. created a Bevacizumab nanoparticle ocular implant to provide a continuous release of the drug.^[^ ^103^ ^]^ This addresses the drawbacks of having to often inject Bevacizumab into the eye to treat CNV. The chitosan nanoparticles carrying Bevacizumab were prepared using an ionotropic gelation method, resulting in optimized nanoparticles with a size of 78.5 ± 1.9 nm, 67.6 ± 6.7% encapsulation efficiency, and loading efficiency of 15.7 ± 5.7%. Release tests indicated an appropriate prolongation of Bevacizumab release from the carriers over two months. Moreover, polysaccharides like alginate, cellulose derivatives, pectin, and xanthan gum are extensively employed in ocular drug delivery due to their distinctive properties of mucosal adhesion, charge, and permeation.^[^ ^104^ ^]^ These characteristics allow them to effectively extend the duration of therapeutic activity and improve the amount of drug that can be absorbed.

Gelatin, renowned for its exceptional biocompatibility and transparency, has been extensively used in the design and production of hydrogel matrices for various tissue engineering applications, notably within the realm of ophthalmology. Wang et al. created a novel eye drop formulation of gelatin nanoparticles combined with the TAK1 inhibitor 5z‐7‐oxozeaenol, demonstrating its pathologic anti‐angiogenic effects without impacting cellular inflammatory activity.^[^ ^105^ ^]^ Tang and colleagues developed a gelatin‐hyaluronic acid (Gel‐HA) based hydrogel, further incorporating polydopamine (PDA) (Figure 4d–g).^[^ ^106^ ^]^ The findings demonstrated that the Gel‐HA‐PDA hydrogel greatly improved the attachment and migration of retinal progenitor cell (RPC) and guided their selective transformation into retinal neurons. This offers hope for the advancement of biomaterials used in RPC transplantation therapies.

Dendritic nanopolymers are a rapidly emerging field beyond micellar NPs. Distinguished by their highly branched structure, these polymers offer numerous surface functional groups, enabling high drug loading, along with high tunability and stability.^[^ ^107^ ^]^ Yang et al. introduced a novel dendritic polyamidoamine (PAMAM) nanocarrier modified with arginine‐glycine‐aspartic acid (RGD) hexapeptide and penetratin (PEN).^[^ ^108^ ^]^ Evaluation of its performance in posterior segment drug delivery indicated a significant distribution in the cornea and retina. These functionalized NPs remained in the posterior segment for over 12 hours following non‐invasive administration, representing a promising non‐invasive drug delivery system for posterior eye diseases. Cho et al. employed intravitreal injection of dendrimer‐conjugated triamcinolone acetonide (D‐TA) in a mouse model of oxygen‐induced retinopathy (OIR) to effectively suppress inflammatory cytokine production, microglial activation, and preretinal neovascularization. This approach led to improvements in neuroretinal and visual function that were impaired by OIR.^[^ ^109^ ^]^

#### 3.3.3 Metal and Metal Compound Nanoparticles

In contrast to organic nanomaterials, metal and metal compound nanomaterials possess unique biomedical properties, high physiological stability, and resistance to degradation. These characteristics make them particularly suitable candidates for treating eye‐related illnesses and delivering medications to the eye. However, they also present certain challenges and limitations, such as poor biodegradability.

**Figure 5:**
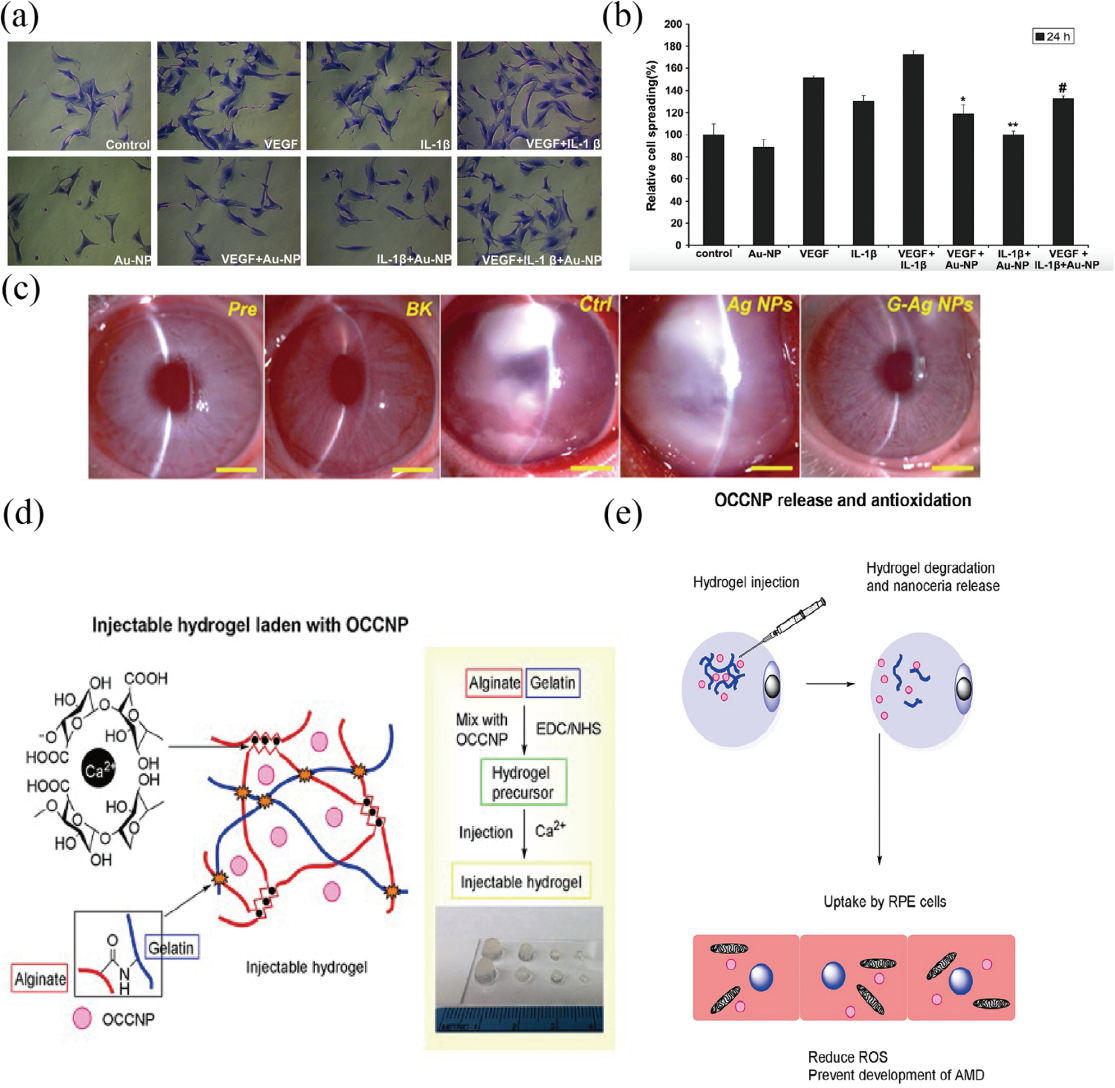
Metal and metal compound nanoparticles. a,b) AuNP's suppression on RPE cell dispersion a) BRPE cells, post‐trypsin treatment, were rinsed in Iscove's Modified Dulbecco's Medium (IMDM) with 10% FBS. Following a 30‐minute pre‐treatment with AuNPs, the cells were cultured on fibronectin‐coated wells (2 × 10^5^ cells per dish) within 35 × 10 mm culture dishes. At the 24‐hour mark, methanol‐fixed and Coomassie‐stained cells displayed inhibited adhesion and spread, both with and without VEGF and IL1‐β exposure. b) Statistical analysis of spread cell percentages across different views illustrated consistent results over three duplicated experiments, expressed as mean ± SEM (*p < 0.05 versus VEGF; **p < 0.05 versus IL‐1β; #*p* < 0.05 for both). Reproduced with permission.^[^ ^111^ ^]^ Copyright 2010, Elsevier. c) Slit‐lamp biomicroscopic pictures were taken of rabbit eyes before the surgery (Pre group) and 3 days after experimentally inducing bacterial keratitis (BK group), followed by intrastromal administration of nanoparticles (Ag NPs or G‐Ag NPs). Reproduced with permission.^[^ ^116^ ^]^ Copyright 2019, Elsevier. d) Schematic representation of the synthesis and e) The mechanism by which antioxidants are delivered and operate in RPE cells using injectable hydrogels loaded with Nanoceria. Reproduced with permission.^[^ ^120^ ^]^ Copyright 2018, Wiley.

Gold nanoparticles (AuNPs) have significant potential in delivering ocular drugs. This is attributed to their distinct anti‐angiogenic and anti‐inflammatory capabilities.^[^ ^110^ ^]^ Karthikeyan et al. experimental findings have verified that AuNPs may efficiently hinder the growth and movement triggered by VEGF and IL‐1β in the retinal pigment RPE by blocking the Src kinase pathway (**Figure** **5a,b**).^[^ ^111^ ^]^ This positions them as effective therapeutic agents for treating eye diseases, such as proliferative vitreoretinopathy. Pereira and colleagues assessed the antioxidative properties of gold nanoparticles in endotoxin‐induced rat uveitis.^[^ ^112^ ^]^ Their findings revealed a reduction in TNF‐α and myeloperoxidase levels following administration of AuNPs, suggesting that gold nanoparticles mitigate inflammation and oxidative damage by interrupting the TLR4‐NF‐κB pathway. Basuki et al. designed an AuNPs‐agarose hydrogel drug delivery system for the “on‐demand” remote release of medications.^[^ ^113^ ^]^ The hydrogel matrix underwent a reversible softening due to the local increase in temperature generated by visible light exposure, which helped control the release of the pre‐loaded drug or protein.

Similar to AuNPs, a range of research indicates that silver nanoparticles (AgNPs) have extensive applications in treating eye diseases. AgNPs, known for their excellent antimicrobial and antioxidative properties,^[^ ^114^ ^]^ are widely used in treating ophthalmic infectious diseases.^[^ ^115^ ^]^ Furthermore, AgNPs also exhibit remarkable potential in combating choroidal neovascularization. Luo and his colleagues produced gelatin‐capped silver nanoparticles (G‐Ag NPs) with a size of ≈14 nm. (Figure 5c).^[^ ^116^ ^]^ They achieved this by encapsulating gelatin molecules onto silver nanoparticles in a controlled environment. The G‐Ag nanoparticles significantly inhibited the growth, movement, and formation of blood vessels in human umbilical vein endothelial cells (HUVEC). In addition, they demonstrated a strong inhibitory effect on the urachus of the chick chorioallantoic membrane and the formation of neovascularization in the cornea of rabbits. These results indicate that AgNPs can be promising candidates for managing various ocular diseases.

Cerium nanoparticles (CeO_2_‐NPs) are endowed with abundant redox‐reactive sites, allowing reversible transitions between trivalent (III) and tetravalent (IV) oxidation states, thus possessing superior redox capabilities and potential for free radical scavenging. Oxidative stress is a common pathophysiological basis for many ophthalmic diseases, including AMD and DR.^[^ ^117^ ^]^ ROS not only directly damages tissues but also promotes pathological neovascularization in the retina and choroid. RP, a genetic degenerative disease mediated by oxidative stress,^[^ ^118^ ^]^ has shown in rodent RP models that CeO_2_‐NPs can provide sustained retinal protection by reducing oxidative stress.^[^ ^119^ ^]^ Recently, soluble CeO_2_‐NPs have been developed. Wang et al. successfully developed an injectable hydrogel filled with oligochitosan‐coated cerium oxide nanoparticles (OCCNPs) alginate, which exhibited biocompatibility, superior antioxidative properties, and anti‐apoptotic capabilities in AMD cell models and a dry AMD mouse model (Figure 5d,e).^[^ ^120^ ^]^ Luo et al. modified hollow cerium dioxide nanoparticles (hCe NPs) by attaching chitosan and ZM241385 and subsequently loaded them with berberine to develop an eye drop formulation for the management of glaucoma.^[^ ^121^ ^]^ This formulation successfully facilitated the opening of tight connections in the corneal epithelium, allowing medication molecules to be delivered to the specific tissues within the eye, exhibited exceptional antioxidative properties, and extended the time to normalize elevated intraocular pressure by 42 times compared to commercial eye drops.

In recent years, the attention‐grabbing Mxene, a novel 2D nanomaterial akin to graphene, has garnered widespread attention. Mxene, a class of 2D transition metal carbides, nitrides, or carbonitrides derived from MAX phases, follows the generic formula of M_n+1_AX_n_, where “M” denotes a transition metal, “A” indicates a group element, and “X” signifies carbon and/or nitrogen. The “A” elements are chemically active, allowing for the selective etching of the “A” layer from the MAX phase using acids such as hydrofluoric acid or NH4HF2. This is followed by ultrasonication to produce the new 2D Mxene material. Like other 2D materials, Mxene possesses a large surface area and exceptional electronic, mechanical, and physicochemical properties.^[^ ^122^ ^]^ These characteristics make it highly applicable in fields like energy storage and catalysis.^[^ ^123^ ^]^ However, in biomedicine, Mxene exhibits superior properties and potential applications compared to other common 2D materials.^[^ ^124^ ^]^ The material is characterized by its outstanding hydrophilicity, enhancing its biocompatibility.^[^ ^125^ ^]^ Additionally, it features multiple surface functional groups.^[^ ^126^ ^]^ These groups are key for the loading of a variety of substances.^[^ ^127^ ^]^

He et al. synthesized a high‐entropy Mxene material, HE‐Mxene, which exhibits outstanding photothermal conversion efficiency and extremely high biocatalytic activity within the second near‐infrared biological window (NIRII).^[^ ^128^ ^]^ It demonstrates superior antibacterial and anti‐biofilm activities both in vitro and in vivo. With single‐layer, HE MXenes + NIRII achieved a bactericidal rate of up to 96.5% against the Gram‐positive drug‐resistant 43 310 strain of Methicillin‐resistant Staphylococcus aureus (MRSA). HE‐Mxene functions as a nanotherapeutic agent by utilizing a technique that enhances its intrinsic oxidase‐mimicking activity in the NIRII range. This permits the efficient treatment of bacterial keratitis (BK) and subcutaneous abscess infections caused by MRSA.

The encouraging findings indicate that nanoparticles made of metal and metal oxides hold promise as strong therapeutic agents for treating eye diseases. Investigating the potentially harmful effects of these substances on the healthy retina and other ocular components is crucial.^[^ ^129^ ^]^ This research is essential to ensure the safety and efficacy of their application in ocular treatments.^[^ ^130^ ^]^

#### 3.3.4 Carbon Nanomaterials

Carbon materials have been extensively studied and widely applied throughout human history, owing to the diverse hybridization states of carbon atoms that have resulted in a plethora of allotropes. The carbon nanomaterials family consists of a diverse range of substances, including 0D fullerenes (C60) and quantum dots (Qds), 1D carbon nanotubes (CNTs), 2D materials like graphene and graphene oxide (GO), as well as 3D structures such as carbon nano‐horns (CNH) and nanodiamonds (ND).

Carbon quantum dots (CDs) are emerging carbon nanomaterials, typically within 10 nm in size. These ultra‐small particles are renowned for their excellent fluorescence properties, water dispersibility (solubility), and ease of synthesis and modification. Their versatility has led to widespread applications in various biomedical engineering fields, including photocatalysis, bioimaging, and biosensing.^[^ ^131^ ^]^ Notably, adjusting their size and surface charge can enhance their corneal permeability, showing promising prospects for applications in ophthalmic diseases, particularly those impacting the back section of the eye.^[^ ^132^ ^]^ CDs likewise exhibit remarkable antiangiogenic properties. In a research conducted by Shereema et al., antiangiogenic outcomes of synthesized CDs were evaluated using the chick chorioallantoic membrane (CAM) assay.^[^ ^133^ ^]^ A 100 µg solution of CDs was administered to the CAM of 4‐day‐old chick embryos. On day 12, photographs were taken to evaluate the density of blood vessels in the CAM. Hemoglobin levels were used as an indicator of vascular density and angiogenesis. The microscopic investigation demonstrated that CAMs treated with CDs had a decrease in vascular density in comparison to the control group (PBS). Hemoglobin levels further supported the antiangiogenic characteristics of CDs. Further analyses revealed that the introduction of CDs successfully reduced the concentrations of angiogenic growth factors (such as VEGF and FGF), VEGFR2, and hemoglobin, suggesting that CDs hinder the process of angiogenesis. Zhao et al. synthesized Graphene Quantum Dots (GQDs) from few‐layer graphene sheets.^[^ ^134^ ^]^ The results indicated that GQDs can effectively inhibit HUVEC proliferation and migration, reduce tube formation and sprouting in HUVECs, and significantly reduce non‐perfused areas and neovascular regions in an OIR model in mice. Amyloid‐beta (Aβ) accumulation in brain tissue is a distinctive degenerative alteration in Alzheimer's Disease (AD). Aβ is a peptide composed of 36–43 amino acids, which is produced by the enzymatic cleavage of amyloid precursor protein (APP) by β and γ secretase enzymes. Recent studies have confirmed that Aβ deposition in the retina is also a potential pathological mechanism for various visual dysfunction diseases, such as vision decline in AD patients, glaucoma, DR, and AMD.^[^ ^135^ ^]^ Recently, Li et al. prepared ultra‐small QDs using the pulsed laser ablation method and found that C‐QDs effectively inhibit the aggregation of Aβ42 peptide, reduce the β‐sheet structure, and decrease the cytotoxicity of Aβ42 peptide in cell models and an AD model of Caenorhabditis elegans CL2006.^[^ ^136^ ^]^

Fullerenes, discovered in 1985, are the third stable allotrope of carbon, following graphite and diamond. Composed of 60 carbon atoms and characterized by sp2 hybridized molecules, fullerenes possess a unique cage‐like spherical structure and non‐polar characteristics.^[^ ^137^ ^]^ Notably, fullerenes exhibit dual properties in terms of ROS, meaning they can generate oxygen under visible light irradiation for photodynamic therapy (PDT) and, due to their rich unsaturated carbon‐carbon double bond structure, can undergo addition reactions with free radicals, acting as antioxidants and ’free radical sponges’. They are not only widely used as anti‐aging agents in cosmetics but also considered as candidate molecules for treating various eye diseases,^[^ ^138^ ^]^ bringing transformative hope to the treatment of these diseases.^[^ ^139^ ^]^ Chen et al. evaluated the antioxidative ability of fullerol in a UVB‐induced corneal injury model.^[^ ^140^ ^]^ Fullerol effectively scavenged ROS (•OH, O_2_•^−^) and RNS (DPPH, ABTS, ONOO−), with efficacy comparable or superior to GSH. It downregulated oxidative stress‐related genes such as NRF2, HO‐1, and γ‐H2AX in corneal epithelial cells, protecting the cornea from UVB damage. Zhuge et al. used fullerol to rescue oxidative stress‐induced senescence in RPE cells, confirming its ability to inhibit cell cycle arrest, apoptosis, autophagy, inflammation, and pigment deposition induced by oxidative stress in RPE cells while enhancing their proliferation and migration capabilities, effects associated with the activation of SIRT1.^[^ ^141^ ^]^

**Figure 6:**
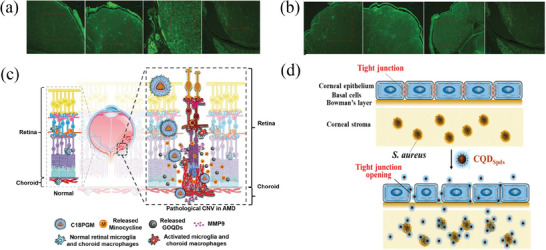
Carbon nanomaterials and quantum dot nanomaterials. a) Eyes injected with CNT‐FITC‐Bio or b) CTN‐FITC‐FA on days 1, 2, and 3 and control eye (×10, DAPID). Reproduced under terms of the CC‐BY license.^[^ ^143^ ^]^ Copyright 2020, published by Knowledge E. c) Schematic illustration depicts the C18PGM as an innovative drug delivery device for the treatment of choroidal neovascularization. Reproduced with permission.^[^ ^147^ ^]^ Copyright 2023, Wiley. d) The utilization of Super‐Cationic Carbon Quantum Dots as an eye drop formulation facilitates the opening of corneal epithelial tight junctions, enabling the effective topical therapy of bacterial keratitis. Reproduced with permission.^[^ ^151^ ^]^ Copyright 2017, American Chemistry Society.

CNTs are a specific form of carbon nanomaterial that has a 1D structure. They can be further categorized into two types: single‐walled carbon nanotubes (SWCNTs) and multi‐walled carbon nanotubes (MWCNTs). CNTs are created through the process of rolling one or several layers of graphene sheets into cylindrical structures. CNTs possess a significant surface area‐to‐volume ratio and aspect ratio, making them suitable for attaching drug molecules either through covalent or non‐covalent bonds. This enables effective loading and release of drugs. Moreover, their needle‐like structure allows them to directly penetrate cell membranes using a “nano‐needle” mechanism, thereby enhancing cellular uptake.^[^ ^142^ ^]^ A study by Demirci et al. validated the penetrative capability of CNT materials in retinoblastoma (**Figure** **6**a,b).^[^ ^143^ ^]^ In this study, transgenic mice received intravitreal injections of CNTs functionalized with isothiocyanate fluorescein and targeted ligand biotin (CNT‐FITC‐Bio) or folic acid (CNT‐FITC‐FA). Higher fluorescence intensity (FI) values were detected, indicating that the CNTs had penetrated the entire retinoblastoma. This suggests their potential as candidate carriers for imaging or treatment of retinoblastoma.

Graphene Family Nanomaterials (GFNs) are a group of materials consisting of one or multiple layers of graphene sheets, which can be either formed into hollow cylinders or organized into flat shapes.^[^ ^144^ ^]^ Members of this family encompass graphene, few‐layer graphene, graphene oxide, reduced graphene oxide, graphene nanosheets, and GQDs, among other variants.^[^ ^145^ ^]^ GFNs have been developed into various formulations, such as eye drops, microneedle patches, and contact lenses to treat ocular disorders.^[^ ^146^ ^]^ The overexpression of matrix metalloproteinase MMP‐9 is a molecular mechanism of inflammation in AMD, especially the wet AMD which is characterized by CNV. Huang et al. designed a medication delivery system utilizing graphene quantum dot materials, C18PGM, which combines GOQD and minocycline (MC) via an MMP9‐sensitive peptide segment (C18P) for responsive release to MMP‐9 (Figure 6c).^[^ ^147^ ^]^ The in vitro results have confirmed the exceptional water solubility and biocompatibility of this drug delivery system. It possesses the capability to release GOQD and MC under the action of MMP‐9, thereby efficiently suppressing the activation and polarization of macrophages and microglia. Consequently, this leads to a reduction in ROS and inflammatory factors, diminishes MMP‐9 and VEGF expression, and inhibits endothelial cell migration and tubule formation, ultimately exhibiting prominent antiangiogenic effects. In a mouse CNV model, C18PGM significantly reduced vascular leakage and area in the CNV region, alleviated macrophage infiltration, and decreased MMP‐9 and VEGF expression, offering a safe and promising therapeutic method for AMD.

#### 3.3.5 QDs

QDs are 0D fluorescent nanomaterials made from semiconductor materials. Typically, these nanomaterials typically consist of elements from either the III‐V group (for example, InP, InAs) or the II‐VI group (such as CdS, CdSe, CdTe, ZnS). They exhibit spherical or quasi‐spherical structure with a diameter between 1 and 10 nanometers.^[^ ^148^ ^]^

Due to quantum size effects, semiconductor QDs display distinctive physical, chemical, and optoelectronic properties that differ significantly from those of their bulk material counterparts. The quantum confinement effect occurs when the size of QDs is similar to or less than the wavelength of their carriers, causing the electrons and holes into a very confined space. This confinement endows QDs with unique quantum characteristics, such as tunable band gaps and discrete energy levels. These features enable QDs to exhibit tunable luminescence colors and high quantum yields. QDs have found application in various fields, including electronics, new energy sources, and biomedicine. For example, quantum dot fluorescent probes offer advantages such as high brightness, wide excitation and narrow emission spectra, long lifespans, and high stability,^[^ ^149^ ^]^ making them suitable for cell imaging, molecular diagnostics, and tumor tracking.^[^ ^150^ ^]^ Furthermore, quantum dot nanomedicines, with their high surface area‐to‐volume ratio, tunable optical properties, and good biocompatibility, can effectively enhance drug bioavailability and hold potential for targeted drug delivery. In ophthalmic drug delivery, their ultra‐small size endows QDs with enormous potential to penetrate ocular barriers.^[^ ^95^ ^]^

Jian and colleagues synthesized supercationic carbon quantum dots (CQDSpds) using spermidine for use as an eye drop formulation (Figure 6d).^[^ ^151^ ^]^ Through immunocytochemical labeling of the protein ZO‐1 associated with tight junctions, that these CQDSpds can efficiently loosen the connections between corneal epithelial cells, thus improving the capacity of drugs to pass through. Additionally, intravenous injection of quantum dot materials can be distributed across all layers of the retina by crossing the BRB.^[^ ^152^ ^]^ Quantum dot materials can function as electrical stimulators for treating retinal disorders. Olson et al. injected silicon‐based QDs into the vitreous body of rats as retinal electrical stimulators, which increased the electroretinogram signals in the retinal photoreceptor cells and effectively prolonged cell survival.^[^ ^153^ ^]^

#### 3.3.6 Hydrogels

**Figure 7:**
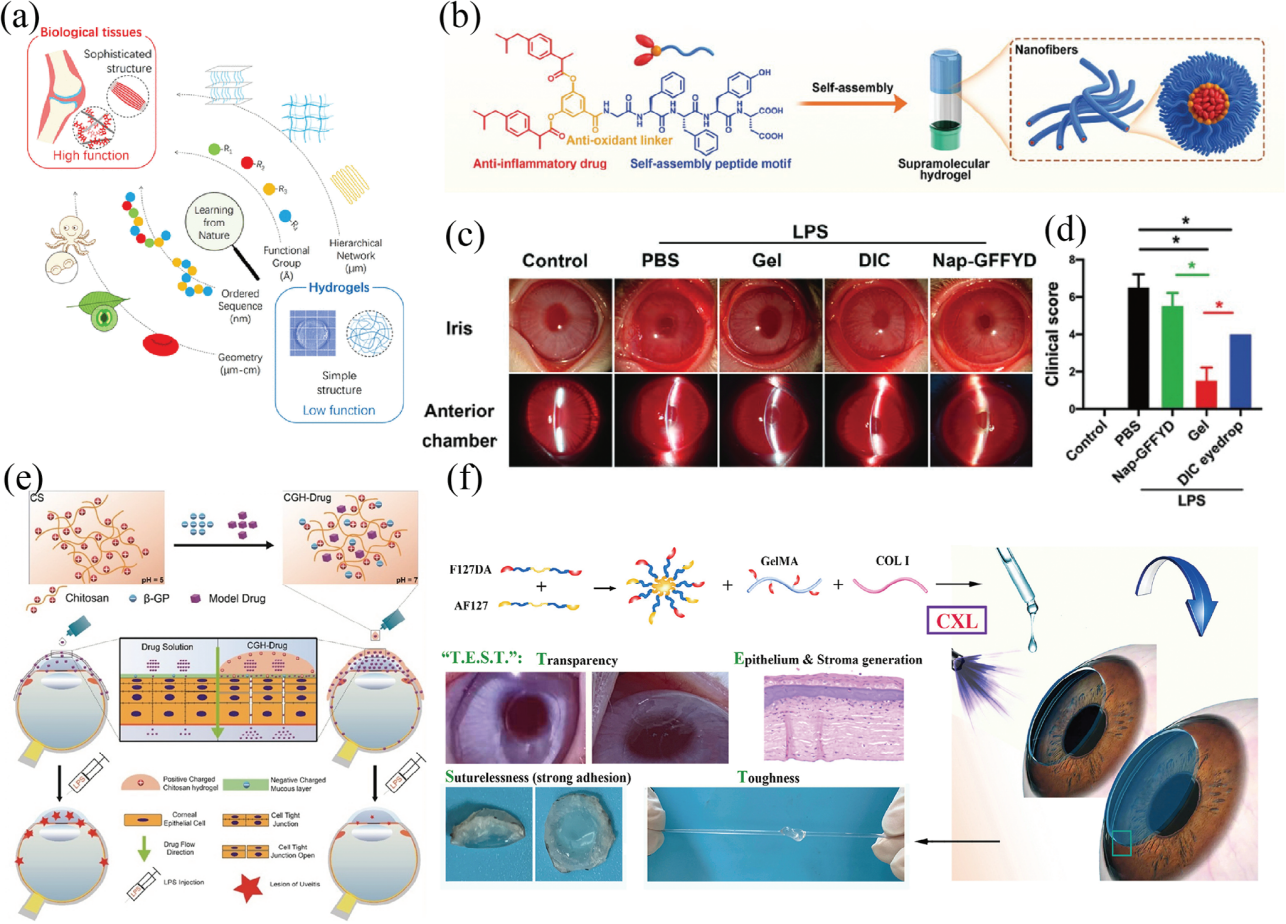
Hydrogel materials with superior biocompatibility for the treatment of ophthalmic diseases. a) The production of bioinspired hydrogels presents both challenges and potential, particularly when considering their physical functions over various length scales. Reproduced with permission.^[^ ^155^ ^]^ Copyright 2020, American Chemistry Society. b–d) The multifunctional supramolecular filament hydrogel demonstrates strong therapeutic effectiveness in the rabbit model of experimental autoimmune uveitis (EIU). b) The self‐assembly of 2IPF‐DHB‐GFFYD to generate a supramolecular hydrogel. c) Slit‐lamp photos (e.g., iris hyperemia, miosis, exudation, hypopyon) of eyes and d) Quantification of inflammatory severity using clinical scores for each group. Reproduced with permission.^[^ ^157^ ^]^ Copyright 2022, Wiley. e) Diagram illustrating the process of the hydrogel eye drops that are loaded with medication, as well as the use of these eye drops for the non‐invasive treatment of uveitis. Reproduced with permission.^[^ ^158^ ^]^ Copyright 2021, Elsevier. f) Light‐cured hydrogel “T.E.S.T” for filling and repairing corneal defects combined with CXL. Reproduced with permission.^[^ ^160^ ^]^ Copyright 2023, Elsevier.

In 1960, Wichterle and Lim achieved a significant milestone in polymer science by synthesizing the first generation of hydrogels using a copolymer of 2‐hydroxyethyl methacrylate and ethylene glycol dimethacrylate.^[^ ^154^ ^]^ Hydrogels are three‐dimensional, crosslinked network materials with high‐water‐content. They can be created by chemically bonding hydrophilic polymer chains together or by polymerizing water‐soluble monomers with crosslinking agents during a polymerization process (**Figure** **7**a).^[^ ^155^ ^]^ These materials exhibit several incomparable properties, such as excellent biocompatibility and biodegradability, tissue‐like flexibility and stretchability, and wide tunability. These characteristics have led to their widespread use in diverse scientific domains, such as tissue engineering, medication administration, and biosensing.^[^ ^156^ ^]^

Deng and colleagues synthesized peptide‐drug conjugates (PDCs) by linking the antioxidant 3,5‐dihydroxybenzoic acid (DHB) and non‐steroidal anti‐inflammatory drugs with self‐assembling peptide segments (GFFYD) through ester bonds, forming a supramolecular hydrogel (Figure 7b–d).^[^ ^157^ ^]^ Owing to the viscoelastic properties of the hydrogel, which effectively resists tear washout, the hydrogel materials provide sustained drug release with a prolonged pre‐corneal residence time, enhancing therapeutic efficacy. The 2IPF‐DHB‐GFFYD supramolecular hydrogel demonstrates combined antioxidant and anti‐inflammatory properties in both laboratory and live animal experiments using a rabbit model of uveitis produced by endotoxins. Chen and others developed a chitosan hydrogel eye drop for non‐invasive treatment of uveitis (Figure 7e).^[^ ^158^ ^]^ This formulation incorporates a hydrogel carrier made of low deacetylated chitosan and β‐glycerophosphate (β‐GP), loaded with the anti‐TNF‐α monoclonal antibody Adalimumab (ADA). The results confirmed that the ADA‐loaded hydrogel eye drops had better penetration and clinical efficacy in treating uveitis compared to ADA alone, enhancing ADA's movement from the eye's outer surface to the underlying choroid layer. This presents a promising approach to minimize adverse effects in the treatment of uveitis without the need for intrusive procedures.

In recent years, in situ hydrogels have gained increasing attention. Hydrogels are divided into pre‐formed and in situ hydrogels. The former are pre‐made in the lab and are gel‐like before application to the eye, while the latter exist as pre‐mixed solutions that turn into hydrogels upon chemical or physical triggering (such as acid‐base reactions, temperature changes, light exposure, etc.).^[^ ^159^ ^]^ Li and colleagues synthesized a photocurable hydrogel named “T.E.S.T” using methacrylated gelatin (GelMA), Pluronic F127 diacrylate (F127DA), aldehyde‐functionalized Pluronic F127 (AF127) co‐assembled dual‐functional micelles, and type I collagen (COL I).^[^ ^160^ ^]^ This “Transparent, supportive, seamless, tough” bioglue is intended for repairing damaged corneas (Figure 7f). Following a 5‐min exposure to UV radiation, the hydrogel demonstrates transparency, exceptional durability, and robust bio‐adhesive characteristics. It is capable of filling and mending corneal defects while simultaneously strengthening the natural cornea. This suggests its potential for treating complex corneal diseases such as severe keratoconus.

#### 3.3.7 Exosomes

Exosomes are extracellular vesicles (EVs) with diameters ranging from 40 to 160 nanometers (averaging around 100 nanometers), formed and released into the extracellular matrix through a series of cellular processes including “endocytosis‐fusion‐excretion.” Nearly all cell types can produce and release exosomes. Initially considered a mechanism for cells to maintain homeostasis by expelling surplus and unnecessary components, recent research has revealed that exosomes can carry components such as nucleic acids, lipids, and proteins depending on their cell of origin, playing a crucial role in regulating intercellular communication and signal transduction. They play a role in a range of bodily functions, both normal and disease‐related.^[^ ^161^ ^]^ These include immunological responses, presenting antigens, cell movement, development, and the spread of tumors.^[^ ^162^ ^]^ This suggests they could be useful in treating and managing numerous disorders.

Exosomes have multiple advantages compared to potentially immunogenic and toxic artificially manufactured exogenous nanocarriers.^[^ ^163^ ^]^ As endogenous natural nanocarriers, they exhibit excellent biocompatibility and bioavailability, with a lower risk of immune rejection, minimum toxicity, and excellent targeting specificity.^[^ ^164^ ^]^ Furthermore, exosomes contain transmembrane and membrane‐anchored proteins, which reinforce endocytosis compared to liposomes and other traditional synthetic nanocarriers, thereby facilitating the transportation of their internal contents.^[^ ^165^ ^]^ Mathew and colleagues documented the beneficial impact of exosomes produced from mesenchymal stem cells (MSCs) on retinal neuronal ischemia.^[^ ^166^ ^]^ After intravitreal injection, MSC‐EVs were absorbed by retinal neurons, ganglion cells, and microglia, and interacted with vitreous components. This prolonged their intraocular presence and reduced cell death and proliferation loss in R28 cells under an in vitro oxygen‐glucose deprivation (OGD) model. Furthermore, MSC‐EVs contributed to an improvement in functional recovery and a decrease in neuroinflammation and apoptosis in a rat model of retinal ischemia reperfusion. The functionality of MSC EVs can be improved through hypoxic preconditioning (HPC) or miRNA engineering, thus enhancing their therapeutic potential. In a separate research, EVs modified for specific functions (known as functionally engineered EVs or FEEs), were used to overexpress miRNA‐424 (referred to as FEE424).^[^ ^167^ ^]^ The results showed a considerable improvement in the neuroprotective and anti‐inflammatory properties of these EVs when tested on retinal cells in vitro. These FEEs were effectively endocytosed by retinal cells, reducing ischemia‐ or inflammation‐induced cell death, proliferation loss, oxygen radicals, and inflammatory factor production in vitro. This enhanced functional recovery after retinal ischemia in vivo and reduced ganglion cell loss, inflammation, and apoptotic gene expression.

## 4 Strategies for Targeted Treatment of Posterior Ocular Diseases

By employing design and modification techniques, a wide range of nanomaterials can efficiently transport therapeutic doses of medications to the posterior section of the eye, thereby improving the drug's capacity to pass through, remain stable, and specifically target the desired areas within the eye. Depending on the therapeutic interventions of retinal diseases and the target points of nanomaterials, the specific mechanisms can be categorized into three main categories: anti‐VEGF therapy, ROS scavenging, and promotion of macrophage polarization.

### 4.1 Anti‐VEGF Therapy

The discovery of VEGF has significantly reshaped our understanding of neovascular diseases in ophthalmology and has offered highly promising treatment approaches. In 2004, Napoleone Ferrara at Genentech, created bevacizumab (pegaptanib), a humanized antibody targeting vascular endothelial growth factor.^[^ ^168^ ^]^ The FDA approved its usage in conjunction with chemotherapy for treating colorectal cancer, making it the first of its kind. In the same year, the FDA also approved an RNA complex (Pegaptanib – Macugen) for treating neovascular AMD.^[^ ^169^ ^]^ The 28‐nucleotide ribonucleic acid “aptamer” assumes a special three‐dimensional structure that selectively attaches to extracellular vascular endothelial growth factor 165, thereby preventing its interaction with receptors. It has shown promising results in the clinical trial. During a therapy period that lasted for more than a year, it effectively decelerated the rate of visual deterioration in patients with AMD. Anti‐VEGF therapy is now widely used as a primary treatment for several eye illnesses affecting the back of the eye. It has shown impressive effectiveness in treating several ailments, such as DR with macular edema and AMD. However, conventional anti‐VEGF therapy often involves repeated intravitreal injections, limiting the bioavailability of the drugs and associated with many adverse reactions and side effects. Addressing the pressing issue of developing and investigating new therapeutic strategies to enhance the effectiveness and safety of anti‐VEGF therapy is crucial.

**Figure 8:**
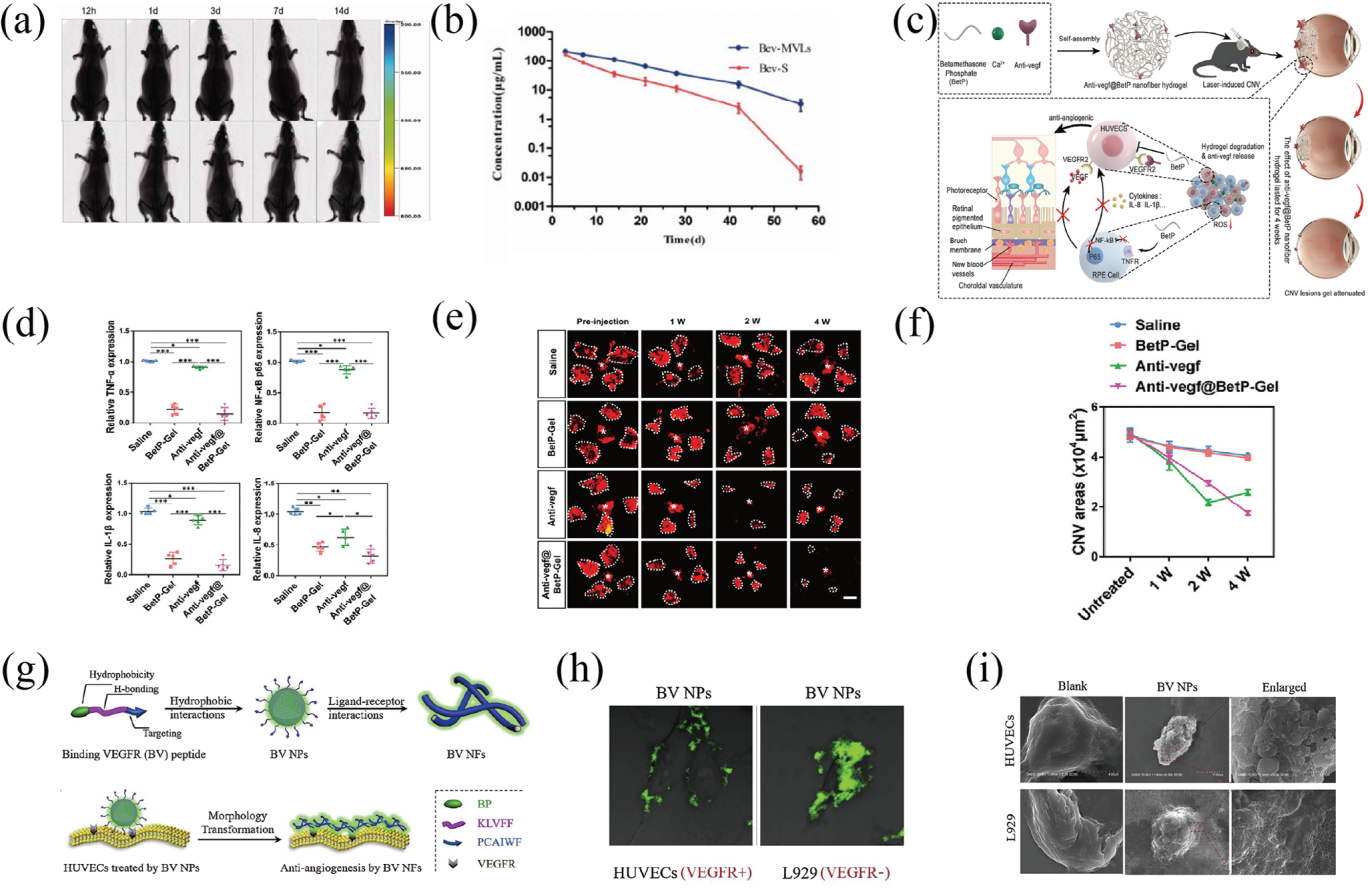
Anti‐VEGF nanomaterials for posterior ocular diseases. a) Rats were subjected to in vivo imaging following intravitreal administration of CB and CB‐MVLs and b) The levels of Bev‐MVLs and Bev‐S were measured in the vitreous humor. Reproduced under terms of the CC‐BY license.^[^ ^173^ ^]^ Copyright 2018, Taylor & Francis. c) The process of creating the Anti‐VEGF@BetP nanofiber hydrogel and its strategy for treating a mouse model of laser‐induced CNV, d) The mRNA levels of TNF‐α, NF‐κB p65, IL‐1β, and IL‐8 in choroidal‐retinal flat mounts reduced by the BetP‐gel, based on qPCR analysis, e) Representative IB4 staining images of individual lesions from the laser‐induced mouse CNV model at the 4th week after intravitreal injection with BetP‐Gel in comparison with saline, anti‐VEGF, and Anti‐VEGF@BetP‐Gel and f) Quantification of the CNV lesions. Reproduced with permission.^[^ ^175^ ^]^ Copyright 2023, Wiley. g) The diagram depicts the process of the binding of VEGFR (BV) peptide with VEGFR and its effect on HUVECs for the inhibition of angiogenesis, h) CLSM images of HUVEC cells and L929 cells incubated with BV NPs (20 mmol L^−1^) for 4 h and i) SEM images of cell surfaces of HUVECs or L929 treated with BV NPs, and untreated cells as blank groups.^[^ ^176^ ^]^ Copyright 2020, Elsevier.

Combining anti‐VEGF drugs with different types of nanoparticle matrices can effectively improve their pharmacokinetics, enhance drug solubility or permeability.^[^ ^170^ ^]^ It can also increase bioavailability, and achieve sustained release.^[^ ^171^ ^]^ Pandit and colleagues used chitosan‐coated PLGA nanoparticle carriers to deliver bevacizumab, with excellent drug loading capacity and encapsulation efficiency (EE).^[^ ^172^ ^]^ The optimized CS‐coated NPs remained on the scleral surface, demonstrating improved permeability and prolonged controlled release, potentially reducing the frequency of administration. Mu and others developed a multivesicular liposome system loaded with Bevacizumab (Bev‐MVLs) for treating laser‐induced CNV (**Figure** **8a,b**).^[^ ^173^ ^]^ Bev‐MVL exhibited a high encapsulation efficiency (80.65%), showing sustained drug release, maintained the structural stability of Bevacizumab, exhibited prolonged vitreous retention in rat ocular imaging, and demonstrated a longer half‐life and higher bioavailability in rabbit pharmacokinetics, effectively inhibiting the formation and progression of CNV, and reducing the thickness of neovascularization.

Combining anti‐inflammatory drugs, antiangiogenic medications, and sustained‐release injection techniques offers promising new perspectives for the treatment of CNV. Liu and colleagues conducted a study where they prepared dexamethasone‐loaded PLGA/PEI nanoparticles (DPPNs).^[^ ^174^ ^]^ These DPPNs were then used to electrostatically adsorb bevacizumab, resulting in the formation of electrostatically‐conjugated bevacizumab‐bearing DPPNs (eBev‐DPPNs). The study sought to assess the effectiveness of these eBev‐DPPNs in inhibiting angiogenesis. The results demonstrated good antiangiogenic effects on HUVECs, effectively inhibiting VEGF secretion. In both in vivo chick embryo chorioallantoic membrane assays and rabbit CNV models, the nanoparticle showed antiangiogenic properties and a reduction in CNV leakage area. Gao and others mixed the steroidal anti‐inflammatory drug betamethasone phosphate (BetP), anti‐VEGF drugs, and calcium chloride to form an injectable hydrogel material (Anti‐VEGF@BetP‐Gel) with a nano‐fiber elastic network structure.^[^ ^175^ ^]^ This hydrogel material was developed for treating wAMD (Figure 8c). This hydrogel material exhibits multiple functions, including anti‐inflammatory, ROS scavenging, and providing prolonged release of anti‐VEGF agents. It effectively encapsulates and releases anti‐VEGF drugs like Ranibizumab. It is biocompatible with ARPE19 cells, resisting hydrogen peroxide‐induced oxidative stress and cellular damage, and reducing the expression of pro‐inflammatory factors such as VEGF, IL‐6, and IL‐8 (Figure 8d). In animal models, it significantly inhibits the formation and proliferation of choroidal neovascularization, improving the pathological state of wAMD (Figure 8e,f).

In addition to the widely used anti‐VEGF therapies like bevacizumab, ranibizumab, aflibercept, and compbercept, there are also other medications being investigated that target the VEGF pathway for treating eye problems in the posterior segment. Wen et al. designed a self‐assembling peptide that targets the VEGF receptor (Figure 8g).^[^ ^176^ ^]^ When dissolved in water, this peptide spontaneously forms a nanotubular structure, with fatty acid chains on the inside and peptide chains on the outside. This distinctive architecture accurately replicates the ligand binding regions of the VEGF receptor, enabling it to attach to the VEGF receptor and impede its interaction with VEGF (Figure 8h,i).

### 4.2 ROS Scavenging

Imbalances in ROS and oxidative stress are common pathophysiological processes in many diseases. In the new century, the concept of “ROS medicine” has gained increasing attention, with researchers focusing on ROS to provide viable therapeutic approaches for inflammation. Significant advancements in nanotechnology have led to major progress in antioxidant treatments. Nanoparticles like carbon (C), cerium (Ce), and polymers possess unique ROS scavenging properties, and many nanoplatforms with ROS‐regulating capabilities have been used in diagnosing and treating posterior segment eye diseases.^[^ ^177^ ^]^ These novel antioxidant nanomaterials perform comparably or even superiorly to traditional antioxidants, such as Vitamin C and N‐acetylcysteine. More importantly, they improve the pharmacokinetics of antioxidant molecules in the body, exhibiting enhanced stability and safety. Antioxidant nanoparticles can be classified into three categories based on their mechanism to scavenge ROS: nanozymes, free radical scavenger nanoparticles, and redox ROS scavenging nanoparticles.^[^ ^178^ ^]^

**Figure 9:**
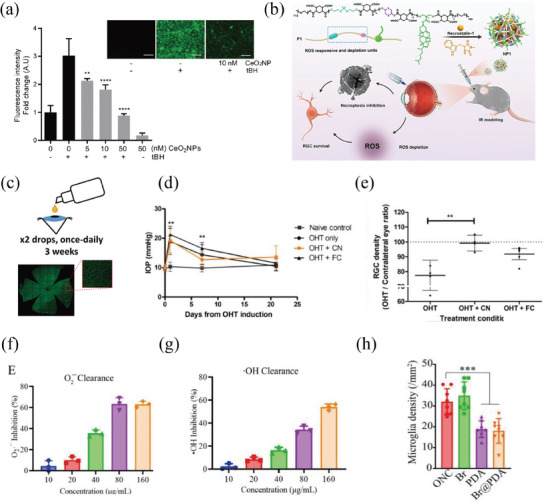
ROS scavenging nanomaterials for posterior ocular diseases. a) ARPE19 cells were treated with CeO2NPs for 24 hours, resulting in a reduction of intracellular ROS levels. Oxidative stress was induced with tBH. Reproduced with permission.^[^ ^180^ ^]^ Copyright 2023, American Chemical Society. b) Schematic illustration of the designed NP1 targeting the cell membranes, inhibiting necroptosis, and depleting ROS in acute glaucoma. Reproduced with permission.^[^ ^182^ ^]^ Copyright 2022, American Chemical Society. c–e) Curcumin nanoparticles applied topically provide in vivo protection for retinal ganglion cell soma against cell loss induced by ocular hypertension. c) Diagram illustrating the in‐vivo experiment method. OHT rats were randomly assigned to one of three groups: no therapy, once‐daily administration of curcumin nanoparticles (CN) eye‐drops, or once‐daily administration of free curcumin (FC) eye drops. The treatment started two days before inducing IOP. Animals were euthanized three weeks later, and their retinas were prepared as flat mounts for labeling with Brn3a. d) The IOP of all animals subjected to OHT showed a substantial increase compared to the first measurement, up until 21 days post‐surgery. There was no disparity in IOP among the different treatment groups for OHT at any given time, indicating that any reported neuroprotective effects were not influenced by IOP. e) Elevated IOP in eyes with OHT only was linked to a decrease in RGC density by ≈23%. Treatment with CN but not FC significantly decreased the loss of RGCs. Reproduced under terms of the CC‐BY license.^[^ ^184^ ^]^ Copyright 2018, published by Nature Publishing Group. f,g) ROS scavenging activities of PDA nanoparticles and the effects of Br@PDA on RGC survival and h) Axon regeneration 30 days after ONC. Reproduced under terms of the CC‐BY license.^[^ ^185^ ^]^ Copyright 2021, published by Biomed Central.

Nanoenzymes are NPs with SOD/catalase (CAT)‐like activities, which are powerful free radical scavengers, such as NPs containing Ce, Pt, Cu elements. Shin and colleagues developed a non‐invasive cerium oxide nanoparticle (CNP) delivery chip (Cerawafer) for regulating ROS in the retina.^[^ ^179^ ^]^ The material alternates redox between Ce3+ and Ce4+, and CNPs synthesized at 0.078, 0.156, 0.312, 0.625, and 1.25 millimolars showed SOD activities of 46%, 68%, 86%, 95%, and 99%, respectively. In vivo, it effectively regulated ROS and reduced VEGF expression in the retina of vldlr−/− mice. Badia and colleagues prepared 3 nm cerium oxide (CeO2 NPs)‐based eye drops for alleviating oxidative stress in the retina and treating AMD (**Figure** **9a**).^[^ ^180^ ^]^ CeO2 NPs protected ARPE19 cells from oxidative stress in vitro, reduced intracellular ROS levels, enhancing the expression of genes related to antioxidants, and impeding the migration and angiogenesis of HUVEC. The antioxidant effects were confirmed in the DKOrd8 mouse model as well. Treatment with CeO2 NPs eye drops led to an augmentation in the thickness of the outer nuclear layer of the retina, a decrease in focal retinal damage, and an enhancement in retinal function.

Materials such as TEMPO and fullerenes, due to their unique chemical structures, are excellent electron acceptors and can capture unpaired electrons from other free radicals, acting as superior radical scavengers.^[^ ^181^ ^]^ Rong and others designed a nanoparticle drug carrier based on polymer P1 (NP1), which encapsulates necrostatin‐1 and releases it in response to ROS (Figure 9b).^[^ ^182^ ^]^ The main chain of NP1 contains thioketone bonds and 1,4‐dithiane units that can consume ROS, while the side chain cholesterol targets the cell membrane. NP1 effectively enters R28 cells, clears ROS in OGD model, inhibits cell apoptosis, improves cell survival, and protects RGCs in the retinal ischemia‐reperfusion (IR) mouse model. It reduces ROS levels, downregulates p‐MLKL and RIPK1 expression, and restores visual function, offering a potential strategy for the treatment of glaucoma.

Common materials for redox reaction‐based free radical scavenging include curcumin nanoparticles, bilirubin nanoparticles, and polydopamine nanoparticles.^[^ ^178^ ^]^ Curcumin, a polyphenolic compound extracted from turmeric, plays a role in regulating biochemical processes related to neurodegenerative and inflammatory diseases, such as oxidative stress, inflammatory response, and β‐amyloid aggregation. Its protective effect on RGCs has been reported, but its clinical use is restricted due to its inadequate solubility and low bioavailability.^[^ ^183^ ^]^ Davis and others developed a novel Pluronic‐F127 stabilized D‐α‐tocopherol polyethylene glycol 1000 succinate Curcumin‐loaded nanocarrier (CN), which dissolves high concentrations of curcumin (4.3 mg mL^−1^) with high encapsulation efficiency (>90%), small particle size (<20 nm), good stability, and sustained‐release properties (Figure 9c–e).^[^ ^184^ ^]^ This material, when tested in R28 retinal cell lines and rodent models, can effectively resist glutamate‐ and cobalt‐induced cytotoxicity, ocular hypertension (OHT) related to glaucoma, and loss of RGCs due to partial optic nerve transection (pONT). It has the potential to be an effective treatment for glaucoma and other eye illnesses that involve damage to the nerves. Lou and colleagues synthesized polydopamine nanoparticles (PDA NPs) that can effectively eliminate various types of ROS, including superoxide anion, hydroxyl radicals, and DPPH radicals (Figure 9f–h).^[^ ^185^ ^]^ PDA nanoparticles reduce ROS levels in cells like HUVECs and N2a, maintain the tight junctions and barrier functions of HUVECs, and inhibit polarization of M1‐type macrophages. In an optic nerve crush (ONC) model, a solitary intravitreal injection of PDA nanoparticles can clear ROS in the retina, significantly reducing the loss of RGCs. This subsequently reduces inflammatory reactions and preserves the barrier function of the retinal vascular endothelium. PDA nanoparticles can deliver Brimonidine (Br@PDA), synergistically reducing the loss of RGCs and promoting axonal regeneration, thereby restoring visual function.

### 4.3 Regulating Macrophage Polarization

Macrophages are essential components of the innate immune system and are involved in the development of various eye disorders. They have gradually become promising therapeutic targets for posterior segment eye diseases, especially those associated with neovascularization. Macrophages respond to internal and external stimuli by polarizing between M1 and M2 phenotypes, fulfilling different physiological roles. M1 macrophages exhibit a pro‐inflammatory phenotype, secreting copious amounts of pro‐inflammatory cytokines, including tumor TNF‐α and IL‐1β, which play a crucial role in combating pathogens and tumor cells. However, an exaggerated M1 response has the potential to cause tissue damage and chronic inflammation, posing significant challenges in specific eye diseases like uveitis and keratitis. Conversely, M2 macrophages predominantly contributed to anti‐inflammatory activities and tissue regeneration. These cells secrete cytokines such as IL‐10 and transforming growth factor beta (TGF‐β), aiding in the suppression of inflammation and accelerating the healing of damaged tissue. In eye diseases such as diabetic retinopathy and AMD, the role of M2 macrophages may offer protective effect. By using inhibitors, antagonists, antibodies, or small molecule drugs, it's possible to reduce or inhibit pro‐inflammatory ocular macrophages or to increase or activate anti‐inflammatory and antiangiogenic ocular macrophages. By using nanocarriers such as nanoparticles, liposomes, and microspheres, drugs or genes can be delivered specifically to targeted ocular macrophages, thereby enabling precise treatment.

**Figure 10:**
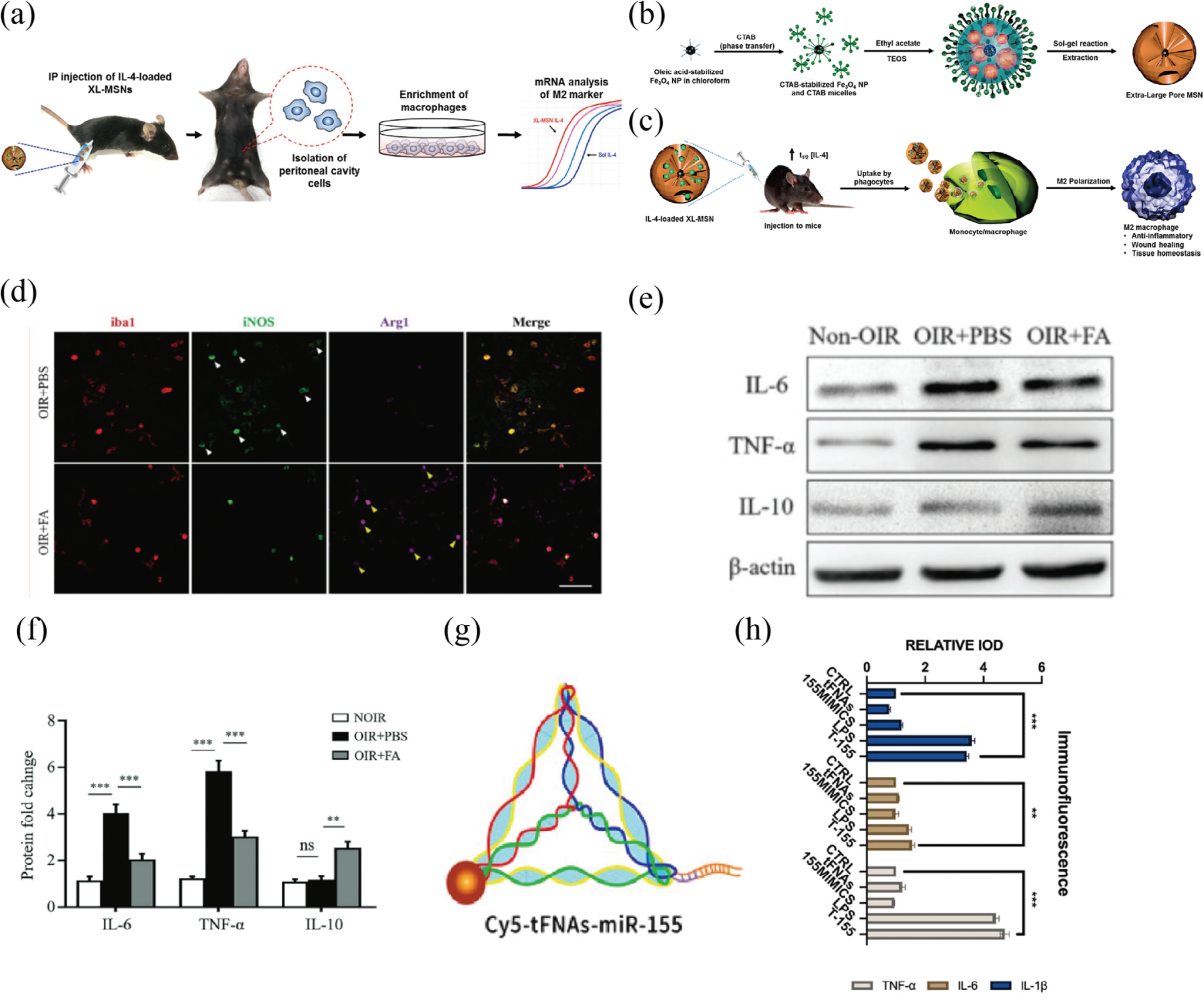
Nanomaterials targeting macrophage polarization for posterior segment ocular diseases. a) Schematic presentation for analysis of M2 polarization by IL‐4‐loaded XL‐MSNs or soluble IL‐4 in vivo. Mice were injected intraperitoneally with soluble IL‐4 (Sol IL‐4) or 180 nm XL‐MSNs (200 µg per mouse) containing IL‐4 (XL‐MSNs IL‐4) at specified IL‐4 dosages (0, 10, 30, 100 ng per mouse). After a period of 3 days, cells located in the peritoneal cavity were collected. Peritoneal macrophages were then isolated by adhering them to culture plates. These macrophages were subsequently used to assess the expression of M2 genes. b) Schematic Presentations of Synthesis of Uniform Mesoporous Silica Nanoparticles with Extra‐Large Pores (XLMSNs). c) Their Application to IL‐4 Delivery for in Vivo M2 Macrophage Polarization. Reproduced with permission.^[^ ^186^ ^]^ Copyright 2017, American Chemistry Society. d–f) FA transformed the phenotypes of microglia/macrophages, shifting them from a pro‐inflammatory state known as “M1” to an anti‐inflammatory one known as “M2”. d) Immunostaining of retinal whole‐mounts revealed that iba1+ microglia/macrophages expressing had elevated levels of iNOS (shown by white arrowheads) rather than Arg1 in the OIR retina. FA treatment resulted in an elevated quantity of Arg1+ iba1+ cells (shown by yellow arrowheads), whereas the amount of iNOS+ iba1+ cells dropped. e,f) The Western blot assay demonstrated that FA therapy resulted in a reduction of IL‐6 and TNF‐a levels, while simultaneously increasing the expression of IL‐10 in the OIR retinas. *n* = 6 mice. Reproduced under terms of the CC‐BY license.^[^ ^189^ ^]^ Copyright 2022, published by Frontiers. g) Schematic structure of T‐155 prepared from tFNA and h) Micro RNA‐155 and and its ability to promote the polarisation of macrophages to the M1 type. Reproduced with permission.^[^ ^192^ ^]^ Copyright 2022, Elsevier.

Kwon and colleagues loaded IL‐4 onto synthesized ultralarge mesoporous silica nanoparticles (XL‐MSNs) to polarize macrophages toward an anti‐inflammatory M2 phenotype (**Figure** **10a–c**).^[^ ^186^ ^]^ XL‐MSN effectively loads IL‐4, displaying good biocompatibility and stability in vitro and in vivo. It effectively promotes macrophage polarization toward the M2 type, expressing more M2 markers such as CD163, CD206, and Arg1, and releasing more M2 factors like IL‐10, TGF‐β, and VEGF. Xu et al. developed a prodrug of epigallocatechin‐3‐gallate for AMD treatment, EGCG‐3‐sulfate (pro‐EGCG), and evaluated its effects on RPE and choroidal neovascularization (CNV).^[^ ^187^ ^]^ In oxidative stress models and laser‐induced CNV mouse models, EGCG‐3‐S effectively inhibits oxidative stress, apoptosis, and autophagy in RPE cells. It reduces the expression of HIF‐1α, VEGF, and VEGFR2, inhibits the activation and infiltration of M1‐type macrophages, and protects RPE cells and CNV. Recent studies have found that microglia/macrophages also undergo similar M1/M2 phenotypic changes as macrophages. Li et al. used flow cytometry to detect the M1/M2 phenotype ratio of microglia/macrophages in the OIR mice's retinas and used western blotting to detect the expression of factors related to M1/M2 phenotypes in the retina.^[^ ^188^ ^]^ The results showed that the M1/M2 phenotype ratio and related factor expression in the retinas of OIR model mice changed with the progression of OIR and were correlated with retinal vascular regeneration.

Modulating the polarization of microglia/macrophages toward the M2 phenotype is a new and promising strategy for treating neovascular disorders. Sun et al. employed Ferulic acid to counteract retinal neovascularization.^[^ ^189^ ^]^ Both in vitro cultured retinal vascular endothelial cells and in vivo hypoxia‐induced retinal disease mouse models demonstrated that FA inhibits ROS/NF‐κB signaling, regulates the polarization of microglia/macrophages from the pro‐inflammatory M1 type to the anti‐inflammatory M2 type, alleviates hypoxia‐induced inflammatory responses, and suppresses hypoxia‐induced retinal neovascularization (Figure 10d–f). Zhang et al. developed a self‐assembling peptide, GFFYE, that transforms the hydrophobic properties of rhubarb into a slow‐release medicinal drug with amphiphilic characteristics.^[^ ^190^ ^]^ They constructed therapeutic nanofibers based on rhubarb (Rh‐GFFYE) for treating retinal ischemia‐reperfusion injury (RIR). Rh‐GFFYE markedly decreases the release of inflammatory cytokines like IL‐1β and IL‐6 and promotes macrophage transition from M1 to M2 phenotype. In rat RIR models, it significantly mitigates the loss of RGCs, increases the thickness of the inner plexiform layer (IPL), improves retinal electrophysiological function, and inhibits ROS, inflammatory factors, and apoptosis‐associated proteins in the retina.

The aforementioned therapeutic strategies promoting macrophage polarization from M1 to M2 types have achieved promising results in certain posterior segment eye disease models. However, recent research has revealed that M2 macrophages not only possess anti‐inflammatory functions but also promote angiogenesis. In the pilot study by Cao et al., they examined the polarization of macrophages in the macular retina and choroid, as well as in CNV membranes (CNVM) of individuals with and without wAMD. They found a higher prevalence of M2 macrophages in wAMD.^[^ ^191^ ^]^ This suggests that macrophages undergo different polarization processes in various disease phenotypes. Qin and colleagues adopted a strategy to promote M1 macrophages polarizing to M2 macrophages to inhibit choroidal neovascularization in AMD. They encapsulated tetrahedral framework nucleic acids (tFNAs) in Micro RNA‐155 to create T‐155 (Figure 10g,h).^[^ ^192^ ^]^ The results showed that after treated with T‐155, the levels of IL1β, TNFα, and IL‐6 in macrophages increased, aligning with the usual control LPS group results, indicating that RAW264.7 cells shifted to the M1 type after T‐155 treatment. Additionally, they used a transwell chamber to co‐culture polarized macrophages with HUVECs and observed angiogenesis in HUVECs post co‐culture. This confirmed that T‐155‐treated macrophages suppressed angiogenesis.

### 4.4 Other Promising Therapeutic Targets

In the previous chapters of this review, we have delved into various strategies and targets for treating posterior ocular diseases, including revolutionary anti‐VEGF therapies, antioxidant approaches, and strategies to regulate macrophage polarization. These methods have demonstrated their significance and efficacy in preclinical research and clinical practice. However, with ongoing scientific advancements and a deeper understanding of the complex mechanisms underlying choroidal retinal diseases, new therapeutic targets are continually emerging, offering additional possibilities and hope for treatment. In this chapter, we will focus on several promising and cutting‐edge therapeutic targets, including fat mass and obesity‐associated protein, matrix metalloproteinases, and fibroblast growth factors. Recent studies have highlighted the potential and innovation of these targets, opening doors to more effective treatment methods.

#### 4.4.1 Fat Mass and Obesity‐Associated Protein (FTO)

Since Jia et al. first reported in 2011 that FTO can reversibly remove methyl groups from N6‐methyladenosine (m6A) in mRNA,^[^ ^193^ ^]^ its role in various diseases, particularly in various cancers, has been extensively studied.^[^ ^194^ ^]^ RNA modifications and epigenetics are among the hottest research areas currently. Like proteins, RNA, a central molecule within the central dogma of molecular biology, undergoes a wide range of chemical modifications, with m6A being the most prevalent mRNA modification in eukaryotes. It plays a vital role in managing biological processes and diseases.^[^ ^195^ ^]^ The m6A modification in cells is reversible and dynamic, with methylation occurring under the action of methyltransferases (termed as “writing”) and demethylation resulting from the action of demethylases (termed as “erasing”). These modification functions through m6A‐binding proteins (known as “readers”) like YTHDF2, with FTO being one of the demethylases of m6A. Located in the cell nucleus, FTO primarily functions to reduce m6A levels through its oxidative demethylase activity, targeting m6A in single‐stranded RNA. Dysregulation of FTO's enzymatic activity can directly affect individual development and disease onset, such as obesity and cancer. Shan et al. discovered FTO's potential in regulating ocular neovascularization in a mouse model of corneal neovascularization. They found that m6A modification levels were reduced in corneal neovascular tissues and endothelial cells, while FTO expression increased. Knockdown of FTO inhibited the formation of corneal neovascularization and endothelial cell proliferation, migration, and tubulogenesis, demonstrating that FTO regulates the FAK gene expression through its m6A demethylase activity, mediated by YTHDF2, thus affecting endothelial cell function and pathological angiogenesis in the eye.^[^ ^196^ ^]^ Furthermore, Tang et al. found that FTO might participate in the inflammatory response and tight junction disruption in RPE cells by modulating the m6A modification of ATF4, thus promoting the development of autoimmune uveitis EAU.^[^ ^197^ ^]^ In a recent study, Wang et al. observed an increase in VEGFA mRNA abundance accompanied by a significant increase in FTO mRNA in choroidal neovascularization induced by laser photocoagulation.^[^ ^198^ ^]^ Utilizing selective inhibitors to block FTO activity in vivo significantly reduced neovascularization, decreased VEGFA protein levels in laser‐treated RPE‐choroid tissue, and inhibited VEGFA release in human ARPE‐19 cells. This finding opens possibilities for FTO‐targeted treatments in angiogenic eye diseases. FTO is considered a potential next therapeutic target with broad prospects following VEGF.

#### 4.4.2 Matrix Metalloproteinases (MMP)

The significant role of MMP‐2 and MMP‐9 in the progression of retinal diseases has been established, generating considerable interest in new therapeutic approaches targeting MMPs. In 2002, Garcia and colleagues verified in animal models that the MMP inhibitor Prinomastat (AGa3340) could effectively inhibit oxygen‐induced retinal neovascularization.^[^ ^199^ ^]^ In 2010, Bhatt and others demonstrated significant protective effects in an experimental rat model of DR using a combined therapy of minocycline and aspirin. The MMP‐2 and MMP‐9 inhibition induced by Minocycline (MINO) was enhanced by Aspirin (ASP), thereby reducing retinal vascular permeability and neuronal apoptosis.^[^ ^200^ ^]^ MicroRNAs have a significant role in regulating several pathophysiological processes, such as angiogenesis, and therapies based on microRNA targeting MMPs have also been applied in treating ocular neovascularization. Gao and colleagues designed a strategy based on microRNA‐195a‐3p to treat neovascularization, an important antiangiogenic factor that can directly target and inhibit the expression of MMP‐2. In a mouse model of choroidal neovascularization, intravitreal injection of miR‐195a‐3p significantly reduced neovascular density, indicating that miR‐195a‐3p induces therapeutic effects against laser‐induced neovascularization.^[^ ^201^ ^]^ Hou et al. used a dual‐luciferase reporter assay to confirm that microRNA‐188‐5p regulates BMCs' function by targeting MMP‐2/13. Its overexpression could reduce the contribution of BMCs to CNV development, potentially representing a novel target for treating CNV‐related diseases.^[^ ^202^ ^]^

#### 4.4.3 Fibroblast Growth Factors

The Fibroblast growth factors (FGFs) family, consisting of a collection of cell signaling proteins, assumes a crucial function in numerous processes, encompassing cellular development. Members from FGF1 to FGF10 bind to fibroblast growth factor receptors (FGFRs). FGF2, also known as basic fibroblast growth factor (bFGF), can be secreted by endothelial cells, smooth muscle cells, and macrophages. It promotes the migration of endothelial cells and the proliferation of smooth muscle cells, thereby facilitating new blood vessel formation.^[^ ^203^ ^]^ Drugs targeting FGFs have been developed and proven effective in ocular diseases. Stahl and others found in an RPE‐induced angiogenesis model that simultaneous inhibition of VEGF and bFGF is an effective antiangiogenic strategy, with better results than inhibiting VEGF alone.^[^ ^204^ ^]^ Jiang et al. validated the pharmacokinetics and ocular distribution of the VEGF/bFGF dual decoy receptor RC28‐E in primate models.^[^ ^205^ ^]^ RC28‐E has high affinity and stability and can effectively inhibit the activities of VEGF and bFGF. Its distribution in the eye is primarily concentrated in the retina, vitreous, and anterior chamber. The half‐life of RC28‐E in the eye is 3.7 days, significantly longer than current market anti‐VEGF drugs.^[^ ^206^ ^]^

## 5 Conclusion

The advancements in nanotechnology‐based drugs and therapeutic strategies have introduced revolutionary progress and new hope into the realm of posterior segment eye disease treatment. In comparison to traditional ocular drug administration methods, nanomaterials, because of their small size, high surface area, adjustable surface properties, and excellent biocompatibility, enhance the penetration, stability, and targeting of drugs within the eye. They enable regulated and sustained continuous release of drugs, thereby reducing the frequency of administration and minimizing systemic side effects. To date, a variety of nanocarriers, including lipid nanoparticles, polymers, carbon nanomaterials, QDs, and so on, have been developed and have successfully delivered drugs to the posterior segment of the eye. Nanomaterials exert therapeutic effects on posterior segment diseases through various mechanisms, such as encapsulating anti‐VEGF agents, scavenging ROS, and promoting macrophage polarization. Some preclinical studies have shown that nanomaterials can effectively inhibit CNV, reduce retinal edema, and protect RGCs and RPE cells. Some promising new targets, such as MMP, FGF, and FTO, also demonstrate great potential. However, nanomaterials still face challenges, including high production costs, difficulties in quality control and storage, and uncertainties in biosafety.

In this review, the barriers and challenges in drug delivery to the posterior segment of the eye are discussed, and numerous examples and proof‐of‐concept studies illustrate the benefits and improvements brought by NPs in treating posterior segment diseases. However, the majority of relevant studies in this field have primarily focused on the cellular and animal levels, yielding significant preclinical results. We anticipate that these promising nanomedicines will be introduced into clinical studies in the future, ultimately benefiting patients. Nanomaterials present a hopeful avenue for ocular drug delivery, offering novel approaches to address posterior segment diseases. Future research should focus on developing more diverse and functional nanomaterials and conducting more in vivo and in vitro experiments to confirm their safety and efficacy.

## Conflict of Interest

The authors declare no conflict of interest.
